# Neuregulin-1 Fosters Supportive Interactions between Microglia and Neural Stem/Progenitor Cells

**DOI:** 10.1155/2019/8397158

**Published:** 2019-04-07

**Authors:** Ghazaleh M. Shahriary, Hardeep Kataria, Soheila Karimi-Abdolrezaee

**Affiliations:** University of Manitoba, Department of Physiology and Pathophysiology, Regenerative Medicine Program, Spinal Cord Research Centre, Rady Faculty of Health Sciences, Winnipeg, Canada R3E 0J9

## Abstract

Microglia play diverse roles in homeostasis and pathology of the central nervous system (CNS). Their response to injury or insult is critical for initiating neuroinflammation and tissue damage as well as resolution of inflammation and wound healing. Changes to the microenvironment of microglia appear to be a key determinant of their phenotype and their role in the endogenous repair process in the injured or diseased CNS. Our recent findings have identified a positive role for neuregulin-1 (Nrg-1) in regulating immune response in spinal cord injury and focal demyelinating lesions. We show that increasing the tissue availability of Nrg-1 after injury can promote endogenous repair by modulating neuroinflammation. In the present study, we sought to elucidate the specific role of Nrg-1 in regulating microglial activity and more importantly their influence on the behavior of neural stem/progenitor cells (NPCs). Using injury-relevant *in vitro* systems, we demonstrate that Nrg-1 attenuates the expression of proinflammatory mediators in activated microglia. Moreover, we provide novel evidence that availability of Nrg-1 can restore the otherwise suppressed phagocytic ability of proinflammatory microglia. Interestingly, the presence of Nrg-1 in the microenvironment of proinflammatory microglia mitigates their inhibitory effects on NPC proliferation. Nrg-1 treated proinflammatory microglia also augment mobilization of NPCs, while they had no influence on their suppressive effects on NPC differentiation. Mechanistically, we show that Nrg-1 enhances the interactions of proinflammatory microglia and NPCs, at least in part, through reduction of TNF-*α* expression in microglia. These findings provide new insights into the endogenous regulation of microglia-NPC interactions and identify new potential targets for optimizing this important crosstalk during the regenerative process after CNS injury and neuroinflammatory conditions.

## 1. Introduction

Microglia play critical roles in the normal and pathologic central nervous system (CNS). In the developing and adult CNS, microglia are involved in homeostasis, phagocytosis of apoptotic neurons, synaptic pruning, release of neurotrophic and growth factors, control of adult neurogenesis, and immune-surveillance [1–6]. Microglia also play pivotal roles in neuroinflammation and innate immune response in neuropathological conditions including neurotrauma, neurodegenerative diseases, stroke, and multiple sclerosis (MS) [7–10]. Following injury, microglia rapidly respond and polarize toward classical proinflammatory or alternative anti-inflammatory or proregenerative phenotypes [11]. Proinflammatory microglia increase their production of proinflammatory cytokines and mediators such as tumor necrosis factor-*α* (TNF-*α*), interleukin-1*β* (IL-1*β*), IL-6, reactive oxygen species (ROS) and nitric oxide (NO), and cluster of differentiation 86 (CD86) receptors, which promote neuroinflammation and tissue damage [12, 13]. Conversely, proregenerative microglia secrete several anti-inflammatory mediators such as transforming growth factor-*β* (TGF-*β*), IL-10, and arginase-1 (Arg-1), which facilitate the resolution of inflammation and support phagocytosis of tissue debris during the regeneration process [14–16]. This evidence indicates the diverse roles that microglia play in endogenous repair mechanisms in the injured or diseased CNS.

Microglia regulate endogenous cell response by influencing fate specification of adult neural stem/progenitor cells (collectively called neural precursor cells (NPCs)) through direct cell-cell interactions or paracrine effects [17, 18]. Lipopolysaccharide- (LPS-) induced proinflammatory microglia block NPC differentiation through expression of TNF-*α* [17], while proregenerative microglia support proliferation, oligodendrocyte differentiation, and mobilization of NPCs through various factors including IL-10, insulin growth factor- (IGF-) I, and TGF-*β*1 [17, 19–22]. Thus, uncovering how the response of microglia is regulated within their microenvironment would allow harnessing their potential for fostering regeneration in the injured and diseased CNS.

We have recently identified a role for neuregulin-1 (Nrg-1) in regulating neuroinflammation and endogenous cell replacement in spinal cord injury (SCI) and focal demyelinating lesions [23–25]. We have shown that Nrg-1 tissue level is acutely and persistently decreased in the injured spinal cord and demyelinating lesions [25, 26]. Nrg-1 is a neuronally derived growth factor that plays essential roles in neural differentiation and myelination [27–29]. We have demonstrated that restoration of the Nrg-1 level promotes proliferation and differentiation of endogenous precursor cells in the injured spinal cord [25, 26]. Intriguingly, our studies have unraveled a positive role for Nrg-1 in regulating T cells and macrophages following SCI [24]. The positive immunomodulatory role of Nrg-1 has also been shown in brain ischemic conditions in which it mitigates neurotoxicity and promotes neuronal survival [30–32]. Our group and others have also shown the ability of Nrg-1 in moderating the response of LPS-induced proinflammatory microglia in culture [23, 32]. Currently, it is unknown how the availability of Nrg-1 in the microenvironment of microglia modulates their effects on the response of NPCs.

In the present study, we sought to determine the direct effects of Nrg-1 on the activity of microglia under normal and proinflammatory conditions and their influence on the regenerative response of NPCs. We induced a proinflammatory phenotype in microglial cultures using injury-relevant proinflammatory cytokines, TNF-*α*, and interferon- (IFN-) *γ*. We demonstrate that Nrg-1 treatment was able to attenuate the expression of proinflammatory mediators in activated microglia. We have also uncovered, for the first time, that Nrg-1 enhances the reduced capacity of proinflammatory microglia for phagocytosis. Availability of Nrg-1 in the environment of proinflammatory microglia attenuated their inhibitory effects on NPC proliferation and enhanced their mobilization, while it was not able to affect the suppressive effects of proinflammatory microglia on NPC differentiation. Furthermore, we show that Nrg-1 promotes the interactions of proinflammatory microglia and NPCs partially by reducing TNF-*α* production in microglia. Taken together, we provide new insights into the role of Nrg-1 in regulating microglial response and importantly their effects on the regenerative capacities of NPCs. Accordingly, this work has identified a new underlying mechanism for Nrg-1 in regulating endogenous repair mechanisms in the injured and diseased CNS.

## 2. Materials and Methods

### 2.1. Experimental Animals

All experimental procedures in the present study were approved by the University of Manitoba Animal Ethics Committee in accordance with the Canadian Council of Animal Care guidelines and policies. We used a total number of 137 postnatal (1–3 days old) and 10 adult enhanced yellow fluorescent protein-tagged (strain name: 129-Tg (ACTB-EYFP) 2Nagy/J) mice for microglia and NPC cultures, respectively, from a colony (CAG-EYFP Tg) maintained at a local facility of the University of Manitoba, Winnipeg, Canada. Mice were housed in standard plastic cages for their newborn pups at 22°C with a 12-hour light/dark cycle. Pelleted food and drinking water were available *ad libitum*.

### 2.2. Microglial Isolation and Culture

Primary microglial cultures were prepared from the cerebral cortex of postnatal EYFP Tg mice (1-3 days of age) as described previously [23, 33, 34]. Briefly, meninges were stripped away from cerebral cortices. Tissues were mechanically dissociated in artificial cerebrospinal fluid solution (aCFS) (containing 124 mM NaCl, 3 mM KCl, 1 mM NaHPO4, 26 mM NaHCO3, 1.5 mM MgSO4, 1.5 mM CaCl_2_, and 10 mM glucose) with 1% penicillin–streptomycin–neomycin (PSN) by 50–60 pipetting strokes. Cells were isolated by centrifugation at 1,000 rpm for 5 min, and the pellet was dissolved in complete DMEM supplemented with 10% fetal bovine serum (FBS) (Gibco) and 1% PSN. Dissociated cells were plated into 75 cm^2^ flasks coated with 1 mg/ml polyethylenimine (PEI) diluted in borate buffer (pH = 8.3). Then, flasks were washed 4 times with phosphate-buffered saline (PBS) (1x) before cell seeding. After 48 h, 8 milliliters of medium was removed to eliminate floating debris and 12 ml of fresh medium was added. No additional medium change was done until the cultures were shaken to separate microglia from any remaining astrocytes (10-14 days). In this method, PEI coating limits the growth of astrocytes and thereby increases the purity and the number of microglia in primary cultures. Of note, we isolated microglia from EYFP mice to be consistent with the strain of the mice that were utilized in our NPC culture.

### 2.3. Microglial Activation

Microglial activation was performed as described in our previous studies [21]. Primary mouse microglia were plated in 24-well plates (1.25 × 10^5^ cells per well) in 50% microglia conditioned media (MCM) (collected and filter-sterilized after shaking) and 50% complete DMEM with 10% FBS. Cells were allowed one day to attach and spread their processes. Media were then changed to serum-free media (SFM) to rule out the influence of serum compositions. One day following serum deprivation, microglia were cotreated with 40 ng/ml of IFN-*γ* and 50 ng/ml of TNF-*α* to induce a proinflammatory phenotype or remained as untreated resting microglia. At 24 h and 72 h after activation, MCM was harvested and stored at –80°C for future experiments. Microglial activation was confirmed using the Griess assay, real-time PCR, Western blot, and immunocytochemical analyses for proinflammatory markers.

### 2.4. Determining Nrg-1 Concentration in Microglial Cultures

In our microglial study, we performed an *in vitro* assay to determine an optimal concentration for recombinant human (rh) Nrg-1*β*1. Of note, rhNrg-1*β*1 used in our study contains the bioactive epidermal growth factor- (EGF-) like domain of all Nrg-1 isoforms (8 kDa; Shenandoah Biotechnology, Warwick, PA). Our dosing assay included 10, 25, 50, and 200 ng/ml of Nrg-1 which are representative of low (10 and 25 ng/ml), medium (50 ng/ml), and high (200 ng/ml) concentrations *in vitro*. The effects of Nrg-1 on proinflammatory microglia were then assessed by Griess assay for nitrite levels in MCM at 24 h and 72 h after Nrg-1 treatment as well as real-time PCR on microglial cell lysate at the 24 h time point. Our dose-response assay identified the efficacy of both 50 ng/ml (as low dose) and 200 ng/ml (as high dose) concentrations of Nrg-1 in various assessments. Our experimental conditions included (1) nonactivated microglia termed as resting microglia (RM), (2) Nrg-1 50 ng/ml-treated resting microglia termed as “RM+Nrg-1 50 ng/ml”, (3) Nrg-1 200 ng/ml-treated resting microglia as “RM+Nrg-1 200 ng/ml,” (4) IFN-*γ*+TNF-*α*-activated microglia termed as “proinflammatory microglia (PM),” (5) Nrg-1 50 ng/ml-treated proinflammatory microglia as “PM+Nrg-1 50 ng/ml,” and (6) Nrg-1 200 ng/ml-treated proinflammatory microglia as “PM+Nrg-1 200 ng/ml.” At 72 h following treatment, MCM were collected and stored at –80°C for future experiments.

### 2.5. Griess Assay for Detection of Nitrite

Nitrite level was measured as a representative of iNOS activity in MCM collected at 24 h and 72 h following microglial activation and Nrg-1 treatment using a Griess assay kit (Promega Corp., USA) according to the manufacturer's instructions. These experiments were performed in phenol red-free media to remove possible color interference in measurement of nitrite levels.

### 2.6. RNA Extraction and Real-Time PCR

One day following microglial activation and Nrg-1 (50 and 200 ng/ml) treatment, microglial RNA was extracted using the TRIzol-based manual method and first-strand cDNA was synthesized using Reverse Transcriptase (Applied Biological Materials Co.). Quantitative analysis of mRNA expression was performed using quantitative PCR (ABI, Perkin-Elmer PE Biosystems, USA), fluorophore SYBR Green I kit (Invitrogen, Canada), and delta-delta Ct method as we previously described [26]. A list of the designed primers in our studies is illustrated in Table 1.

### 2.7. Western Blot Analysis for Cytokine Expression in Microglia Conditioned Media

For Western blot analysis, MCM was concentrated 30 times using a centrifugal column with a molecular weight cutoff at 3 kDa (VWR). The concentration of each sample was measured using the modified Lowry assay. The blocking and antibody solution contained 5% nonfat milk or bovine serum albumin (BSA) in Tris-buffered saline with 0.5% Tween-20 (TBST). A list of the antibodies used in immunoblotting is provided in Table 2. Specificity of the used antibodies in detecting the protein of interest was verified by its specified molecular weight. Fifty *μ*g of MCM, cell or tissue lysate, 100 ng/ml and 1 *μ*g/ml of recombinant TNF-*α*, and 50 ng/ml of recombinant IFN-*γ* were loaded onto SDS-PAGE gels and transferred onto nitrocellulose membranes (Bio-Rad). To control for equal protein loading, the membranes were then stained with Ponceau S staining. After washing the membrane, it was blocked in 5% nonfat milk or BSA in Tween Tris-buffered saline (TTBS) for 1 h at room temperature and then incubated with primary antibodies overnight at 4°C. After one day, the membrane was washed and incubated with horseradish peroxidase- (HRP-) conjugated goat anti-mouse or anti-rabbit secondary antibodies (1 : 4000; Bio-Rad) for 1 h at room temperature. For developing the membrane, it was incubated in enhanced chemiluminescent immunoblotting detection reagents (FroggaBio, Canada) according to the kit's instructions. Densitometry analysis of the immunoreactive bands was then performed using NIH ImageJ software. For MCM, the protein densitometry value was then normalized to the total amount of protein in that lane determined by Ponceau S staining. Values for cell and tissue lysate were then normalized to actin or GAPDH to control for equal protein loading. The bands on the same membrane were quantified and normalized to the control condition.

### 2.8. Microglial Phagocytosis Assay

Microglial phagocytosis assay was performed as we and others described previously [21, 35]. One *μ*l of green fluorescent latex beads with 1 *μ*m diameter (Sigma, L1030) was added to 5 *μ*l of FBS and was kept in an incubator for 1 h. The mixture of FBS and beads was then diluted in microglial SFM and added to the cell culture at the final concentration of 0.01% (*v*/*v*). After 1 h, the medium was removed and cells were washed one time with PBS (1x). The cells were fixed with 3% paraformaldehyde (PFA) and then stained with 4′,6-diamidino-2-phenylindole (DAPI) and imaged. For quantitative analysis, the number of DAPI+ cells containing fluorescent beads was counted. Fluorescent bead engulfment was confirmed by Z-stacking and colocalization of cells positive for ionized calcium-binding adaptor molecule 1 (Iba-1) with a green fluorescent signal emitted from the beads.

### 2.9. Microglial Mobility Assay

Microglial mobilization was performed as described in previous studies by our group and others [16, 21]. Microglia were plated onto an uncoated 24-well plate in SFM. One day after plating, microglia were either induced into a proinflammatory phenotype by IFN*γ*+ TNF-*α* or remained as nonactivated resting microglia. At 24 h after activation, resting and proinflammatory microglia were collected, centrifuged, and plated onto poly-D-lysine- (PDL-) (0.1 mg/ml) coated polycarbonate Transwell culture inserts (Corning, 30,000 cells per Transwell) in SFM in a 24-well plate. Nrg-1 (50 and 200 ng/ml) was added to the bottom chamber. Cells were then incubated for 16 h at 37°C to allow for their mobilization to the bottom chamber. After a 16 h mobilization, cells were fixed with 3% PFA for 20 min and stained for DAPI (1 : 5000). The remaining cells on the upper side of the Transwell were gently scraped off with a cotton swab. Cells mobilizing to the bottom side of the Transwell membrane were then visualized by DAPI. The total number of DAPI+-mobilized microglia was determined by taking eight images at 40x magnification.

### 2.10. Immunocytochemistry, Imaging, and Analysis

For immunocytochemistry, cultures were fixed with 3% PFA for 20 min at room temperature and washed with PBS (1x). Cells were incubated in a blocking solution containing 5% nonfat milk, 1% BSA, and 0.5% Triton X-100 in 0.1 M PBS for 1 h at room temperature. Cultures then underwent an immunostaining procedure as previously described [21]. For 5-bromo-2′-deoxyuridine (BrdU) immunodetection, prior to blocking, slides were incubated in 2 N HCl and 0.5% Triton for 30 min at 37°C and then washed with 0.1 M sodium borate in PBS for 10 min. After blocking, the slides were incubated with primary antibodies overnight and secondary antibodies were added as we previously described (Table 2) [21]. For immunocytochemistry quantification, 8-10 separate fields which contain an average of 300 cells for each condition were randomly imaged at 20x magnification using a Zeiss Imager 2 epifluorescence microscope. For each condition, first the total number of DAPI-positive cells was assessed, and then the number of cells which were positive for nestin, glial fibrillary acidic protein (GFAP), NG2 or BrdU, and DAPI was counted. Under each experimental condition, the percentage of abundance of each cell type was calculated by dividing the number of positive cells for a specific marker by the total number of DAPI+ cells. For a relative comparison, values were then normalized to control condition.

### 2.11. Isolation and Culture of Adult Neural Precursor Cells

Isolation of adult NPCs was performed from the subventricular zone (SVZ) of EYFP Tg (strain name: 129-Tg (ACTB-EYFP) 2Nagy/J) mice (6–8 weeks old) as we extensively utilized in our previous studies [25, 36]. Briefly, mice were euthanized by decapitation after being deeply anesthetized with 40% isoflurane and 60% propylene glycol in a bell jar. Under sterile conditions, the brain was then excised and transferred to aCFS solution. Subventricular zones (SVZs) of each brain were dissected and digested with 1 ml of enzyme mix (1.25 mg/ml trypsin, 0.6 mg/ml hyaluronidase, and 0.13 mg/ml kynurenic acid; Sigma) at 37°C for 45 min. Quenching of trypsin activity was performed using three volumes of inhibitor solution (1 mg/ml; Sigma). Cellular components were then spun for 5 min at 1,500 g, and the pellet was resuspended in the growth medium. Resuspended cells were then plated onto uncoated tissue culture flasks (BioLite, Fisher Scientific) in a final volume of 10 ml of SFM containing Neurobasal-A medium (Invitrogen), 30% glucose, 7.5% NaHCO_3_, 1 M HEPES, 10 mg of transferrin, 2.5 mg of insulin, 0.96 mg of putrescine, 1 *μ*l of selenium, 1 *μ*l of progesterone, 1% L-glutamine, and 1% PSN, as well as growth factors (1 *μ*g of FGF2, 2 *μ*g of EGF, and 200 *μ*g of heparin). The neurospheres generated were passaged weekly by mechanical dissociation in growth media. SFM plus growth factors will be referred to as “growth media” in the text.

### 2.12. Assessment of the Effects of Microglia Conditioned Media on NPC Proliferation

NPC proliferation assay was performed as we described previously [21]. NPC neurospheres were dissociated into single cells and plated onto Matrigel-coated multichamber glass slides (25,000 cells per chamber) (Lab-Tek II) in growth media. At 24 h following cell seeding, media were changed to 50% fresh NPC SFM and 50% MCM (collected at the 72 h time point). For BrdU assay, microglia were activated and treated with Nrg-1 in SFM to rule out the effect of serum on NPC proliferation. The experimental conditions were as follows: (1) control media (50% NPC SFM+50% incubated microglia SFM), (2) RMCM (50% NPC SFM+50% RMCM), (3) RM+Nrg-1 50 ng/ml CM (50% NPC SFM+50% Nrg-1 50 ng/ml-treated RMCM), (4) RM+Nrg-1 200 ng/ml CM (50% NPC SFM+50% Nrg-1 200 ng/ml-treated RMCM), (5) PMCM (50% NPC SFM+50% PMCM), (6) PM+Nrg-1 50 ng/ml CM (50% NPC SFM+50% Nrg-1 50 ng/ml-treated PMCM), and (7) PM+Nrg-1 200 ng/ml CM (50% NPC SFM+50% Nrg-1 200 ng/ml-treated PMCM). Fresh and 72 h preincubated Nrg-1 and fresh and 72 h incubated IFN-*γ*+TNF-*α* combination were also used as controls at their defined concentrations. TNF-*α*-neutralizing antibody (NA) (R&D Systems, MAB4101, 18 *μ*g/ml) was used to assess the overall effect of TNF-*α* on NPC properties. Normal mouse immunoglobulin G (IgG) (Santa Cruz Biotechnology, sc-2025, 18 *μ*g/ml) was also used as control to assess the specificity of TNF-*α* NA function. For assessing NPC proliferation, BrdU (20 *μ*M, Sigma) was added to the cultures 4 h before processing NPCs for immunocytochemistry as described above.

### 2.13. Assessment of the Effects of Microglia Conditioned Media on NPC Differentiation

NPC differentiation assay was performed as described in our previous studies [21, 37]. Dissociated NPCs were plated onto Matrigel-coated multichamber glass slides (15,000 cells per chamber) (Lab-Tek II) in growth media. At 24 h following cell plating, media were changed to 50% fresh NPC SFM and 50% MCM. NPC differentiation was performed in 2% FBS for 7 days and assessed using immunocytochemistry against the NPC specific marker nestin, the astrocyte-specific marker GFAP, and the OPC-specific marker NG2.

### 2.14. Assessment of the Effects of Microglia Conditioned Media on NPC Mobilization

Dissociated NPCs were seeded over the right-sided well in the upper compartment of each microdevice (Ananda), which was placed on a PDL-coated dish (5000 cells per well) (Corning) in NPC SFM. Cells were allowed to migrate to the lower compartment through capillaries for 20 h. Lower compartments contained (1) microglial SFM as the baseline control (control media), (2, 3) fresh and 72 h preincubated Nrg-1 50 ng/ml, (4, 5) fresh and 72 h preincubated Nrg-1 200 ng/ml, (6) RMCM, (7) RM+Nrg-1 50 ng/ml CM, (8) RM+Nrg-1 200 ng/ml CM, (9) PMCM, (10) PM+Nrg-1 50 ng/ml CM, or (11) PM+Nrg-1 200 ng/ml CM. Incubated Nrg-1 (50 and 200 ng/ml) was added to the lower compartment to eliminate the possibility of Nrg-1 effects per se on NPC mobilization. Addition of more medium to the wells of the upper compartment makes a gradient slope that directs movement of NPCs downward the lower compartment through capillaries. Mobilized cells were imaged and counted in capillaries and the lower compartment which connects two wells in each microdevice.

### 2.15. Statistical Analysis

Using SigmaStat Software (4.0), we performed one-way analysis of variance (ANOVA) followed by the Holm-Sidak post hoc test in all immunocytochemistries. The Student *t*-test was used when two groups were compared. The data was reported as means ± standard error of the mean (SEM). *p* ≤ 0.05 was considered statistically significant.

## 3. Results

### 3.1. Activation of Primary Microglial Cultures

**Figure 1:**
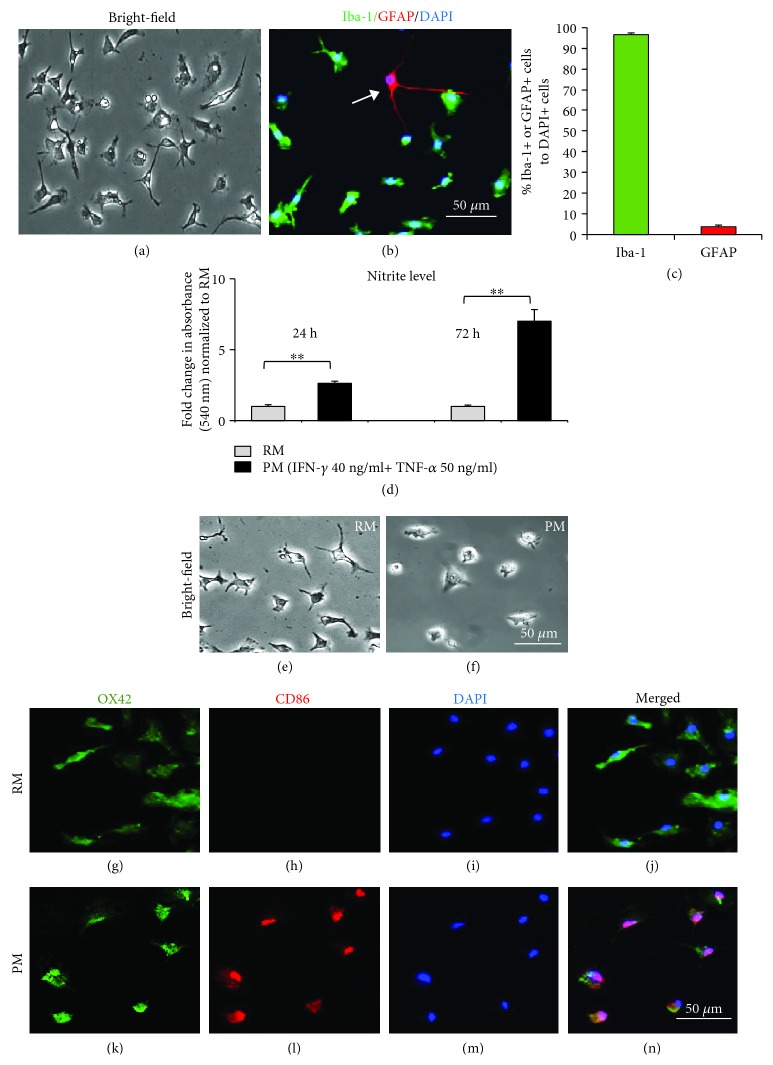
Characterization and activation of primary postnatal microglial cultures. (a) The bright-field microscopy shows resting microglia with ramified morphology and rare presence of astrocytes. (b, c) Quantitative analysis of Iba-1+/DAPI+ microglia and GFAP+/DAPI+ astrocytes revealed that the number of microglia exceeds over 98% with less than 2% contaminating astrocytes confirming the high purity of our microglia cultures. Astrocytes were rarely detected in microglia culture. White arrow in (b) shows one GFAP+/DAPI+ astrocyte that was found in our microglial culture. (d) Activation of microglia was confirmed by Griess nitrite assay. We found a significant increase in nitrite production in the MCM of proinflammatory cultures under cotreatment of 40 ng/ml of IFN-*γ* and 50 ng/ml of TNF-*α*, when compared to resting condition at 24 h and 72 h time points. (e, f) Bright-field images show resting microglia with branches in nonactivated cultures and round-shaped microglia in proinflammatory cultures. (g-n) Coimmunostaining of microglia (OX42) with CD86 antibody showed the induced expression of CD86 in proinflammatory microglia as compared to resting microglia with no detectable signal for CD86. The data represent mean ± SEM, ^∗∗^ *p* < 0.01, *N* = 3 independent cultures, Student *t-*test.

In this study, we utilized a well-established primary culture of postnatal mouse cortical microglia. We assessed the purity and quality of these microglial cultures. Our bright-field microscopy confirmed the presence of resting microglia with ramified morphology (Figure 1(a)). To determine the purity of microglial cultures, we performed double-labeling immunocytochemistry for the microglia-specific marker, Iba-1, and the astrocyte-specific marker, GFAP. DAPI counterstaining was also used to identify nuclei. Quantitative analysis of Iba-1+/DAPI+ cells verified the presence of 98% microglia in these cultures with only 2% of GFAP+/DAPI+ astrocytes (*N* = 4 independent cultures, Figures 1(b) and 1(c)). We next confirmed the expression of all Nrg-1 receptors in our microglial culture using coimmunostaining of OX42 (a known marker for microglia) with ErbB2, ErbB3, and ErbB4 (Supplementary Figures 1a–1i). In agreement with previous studies [38], these immunocytochemical assessments confirmed the responsiveness of mouse-derived microglia to Nrg-1 treatment.

To study the effects of Nrg-1 on the proinflammatory response of microglia, we induced a proinflammatory phenotype in primary microglia by a combination of IFN-*γ* and TNF-*α*, two relevant cytokines involved in activation of microglia in neuroinflammation after CNS injury or disease [39, 40]. We verified microglial activation by assessment of nitrite levels in MCM of control microglia (nontreated resting microglia) and proinflammatory microglia cotreated with IFN-*γ* and TNF-*α*. Our Griess assay showed a significant 2.6- and 7-fold increase in nitrite production in PMCM compared to RMCM after 24 h and 72 h, respectively (*p* < 0.01, Student *t*-test, *N* = 3 independent cultures) (Figure 1(d)). Induced nitrite level is a known property of proinflammatory microglia that implicates them in oxidative stress and neurotoxicity [41, 42]. We also verified activation by assessing microglial morphology using bright-field microscopy. Our observations confirmed microglial activation by transitioning from resting ramified morphology with branches in nontreated primary cultures (nonactivated microglia) to a round-shaped microglia in (activated) proinflammatory cultures (Figures 1(e) and 1(f)). Furthermore, activation of mouse microglia was confirmed by induced expression of CD86, a proinflammatory microglia-specific marker, in IFN-*γ*- and TNF-*α*-treated microglia using coimmunostaining for OX42 and CD86 (Figures 1(g)–1(n)). After these verifications, we employed this culture system to examine the effects of Nrg-1 bioavailability on proinflammatory microglia. We will refer to IFN-*γ*- and TNF-*α*-cotreated microglia as “proinflammatory microglia (PM)” and to nontreated microglia as “resting microglia (RM)” throughout this study.

### 3.2. Nrg-1 Attenuates Proinflammatory Properties of Activated Microglia

**Figure 2:**
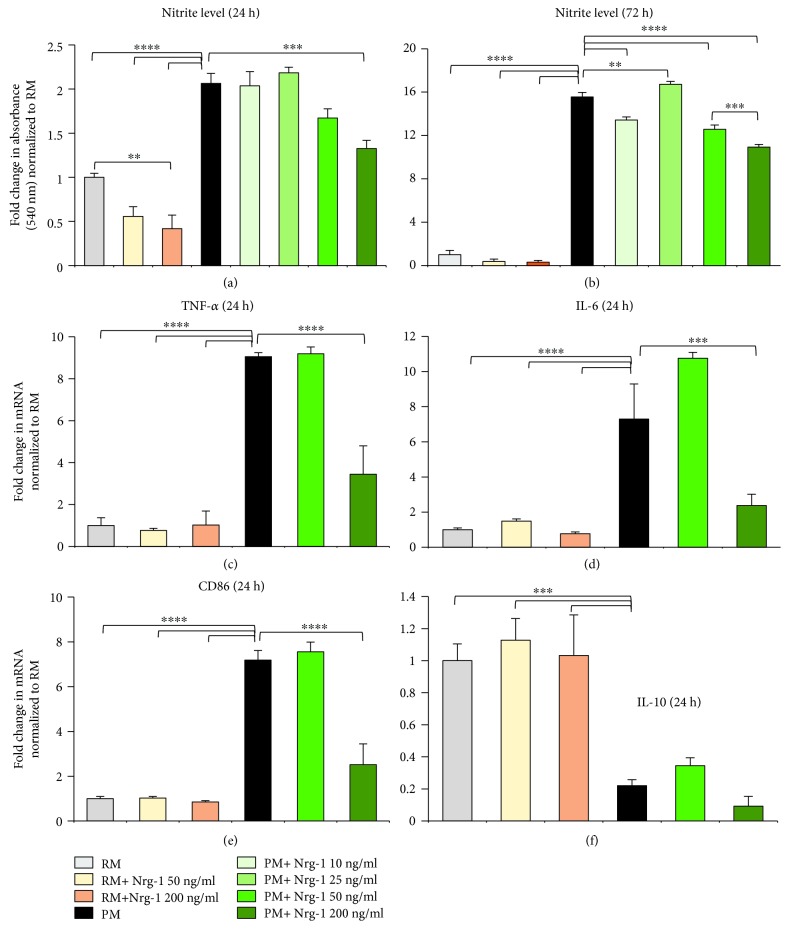
Nrg-1 treatment attenuates nitrite production and expression of proinflammatory cytokines in activated microglia. (a, b) Microglial activation significantly increased nitrite production in MCM as compared to resting microglial cultures. Four concentrations of Nrg-1 were assessed at the 24 h and 72 h time points. Griess assay identified that the most effective concentrations of Nrg-1 in attenuating nitrite production by proinflammatory microglia were 50 ng/ml and 200 ng/ml. (c-e) Microglial activation significantly increased the expression of TNF-*α*, IL-6, and CD86 transcripts at the 24 h time point. Nrg-1 treatment at 200 ng/ml significantly attenuated the induced expression of TNF-*α*, IL-6, and CD86, while Nrg-1 50 ng/ml had no effect on the transcript levels. (f) Analysis of IL-10 transcript levels indicated that activation caused a significant reduction in IL-10 transcript expression in microglia. However, Nrg-1 at both concentrations did not recover the suppressed mRNA level of IL-10. Results were normalized to the mRNA level of H2afz as a housekeeping gene prior to the subsequent normalization to the control values. The data represent mean ± SEM, ^∗∗^ *p* < 0.01, ^∗∗∗^ *p* < 0.001, and ^∗∗∗∗^ *p* < 0.0001, *N* = 3 − 4 independent cultures, one-way ANOVA, followed by the Holm–Sidak post hoc test.

Modulating microglial activation has been a promising immunomodulatory strategy for CNS neuroinflammatory disorders [21, 23]. Here, we investigated whether bioavailability of Nrg-1 at the time of microglial activation can influence their phenotype. First, to determine the best concentration of rhNrg-1*β*1 in our purified mouse microglial cultures, we treated resting and proinflammatory microglia with various concentrations of Nrg-1 including 10 ng/ml, 25 ng/ml (low), 50 ng/ml (medium), and 200 ng/ml (high). At 24 h post-Nrg-1 200 ng/ml treatment, there was a significant 60% decrease in nitrite levels in the MCM of Nrg-1-treated resting microglia compared to nontreated resting conditions indicating that Nrg-1 200 ng/ml reduced the baseline level of nitrite in resting microglia (*p* < 0.01, one-way ANOVA, *N* = 3 independent cultures) (Figure 2(a)). Activation of microglia induced nitrite levels significantly by 2 folds compared to resting microglia at 24 h (*p* < 0.0001, one-way ANOVA, *N* = 3 independent cultures). Our Nrg-1 concentration study showed that 200 ng/ml of Nrg-1 resulted in a significant 36% reduction in nitrite levels in PMCM (*p* < 0.001, one-way ANOVA, *N* = 3 independent cultures) (Figure 2(a)). There was no change in the nitrite level in MCM of proinflammatory microglia treated with 50 ng/ml, 10 ng/ml, and 25 ng/ml when compared to nontreated proinflammatory cells (*p* > 0.05, *N* = 3 independent cultures) (Figure 2(a)). At the 72 h time point, microglial activation resulted in a more pronounced and significant 15.5-fold induction in nitrite production when compared with nonactivated resting condition (*p* < 0.0001, one-way ANOVA, *N* = 3 independent cultures) (Figure 2(b)). Treatment of proinflammatory microglial cultures with 50 ng/ml (19.2%) and 200 ng/ml (30%) of Nrg-1 significantly attenuated nitrite production at the 72 h time point as compared to proinflammatory nontreated microglia (*p* < 0.0001, one-way ANOVA, *N* = 3 independent cultures) (Figure 2(b)). There was a significant increase in the production of nitrite when proinflammatory microglia were treated with 25 ng/ml of Nrg-1 (*p* < 0.01, one-way ANOVA, *N* = 3 independent cultures) (Figure 2(b)). Considering the outcomes of these studies, the best concentration for Nrg-1 was determined to be 50 ng/ml (as a low dose) and 200 ng/ml (as a high dose) for our subsequent experiments.

Activated microglia are involved in neuronal injury and death by producing proinflammatory cytokines and mediators [43, 44]. Microglia also act as antigen-presenting cells (APCs) through the expression of costimulatory molecules (CD86 receptors), which activate other immune cells [45]. Accordingly, we sought to determine whether Nrg-1 at low and high concentrations can modulate microglial expression of proinflammatory cytokines, TNF-*α* and IL-6, anti-inflammatory cytokine, IL-10, and CD86 receptor at 24 h after activation and Nrg-1 treatment. Quantitative real-time PCR revealed a significant increase in mRNA expression of TNF-*α* (9 folds) and IL-6 (7.2 folds) in proinflammatory microglia compared to rather low basal levels of these cytokines in resting microglia (*p* < 0.0001, one-way ANOVA, *N* = 4 independent cultures) (Figures 2(c) and 2(d)). Interestingly, Nrg-1 at 200 ng/ml significantly attenuated the transcript levels of TNF-*α* (62%) and IL-6 (67.5%) in proinflammatory microglia in relation to nontreated proinflammatory microglia (*p* < 0.0001 and *p* < 0.001, one-way ANOVA, *N* = 4 independent cultures) (Figures 2(c) and 2(d)). In addition, Western blot analysis of MCM showed that Nrg-1 200 ng/ml significantly attenuated the induced protein expression of pro TNF-*α* in proinflammatory microglia, which further confirmed the real-time PCR result (*p* < 0.001, one-way ANOVA, *N* = 4 independent cultures) (Supplementary Figures 2a and 2b). We also found a significant 7.2-fold increase in the mRNA level of CD86 in proinflammatory microglia, which was significantly reduced by 65% with Nrg-1 treatment at 200 ng/ml compared to nontreated proinflammatory microglia (*p* < 0.0001, one-way ANOVA, *N* = 4 independent cultures) (Figure 2(e)). Analysis of the IL-10 transcript level indicated that activation significantly reduced (78%) the IL-10 expression in pure microglial cultures (*p* < 0.001, one-way ANOVA, *N* = 4 independent cultures) (Figure 2(f)). However, Nrg-1 had no significant effect on IL-10 expression in resting or activated microglia (*N* = 4 independent cultures) (Figure 2(f)). Western blot analysis of MCM also showed that Nrg-1 had no apparent effect on the expression of arginase-1 (Arg-1), another marker for proregenerative and anti-inflammatory microglia (Supplementary Figure 2c).

### 3.3. Nrg-1 Treatment Restores the Reduced Phagocytic Ability of Proinflammatory Microglia with No Effects on Their Proliferation and Mobility

**Figure 3:**
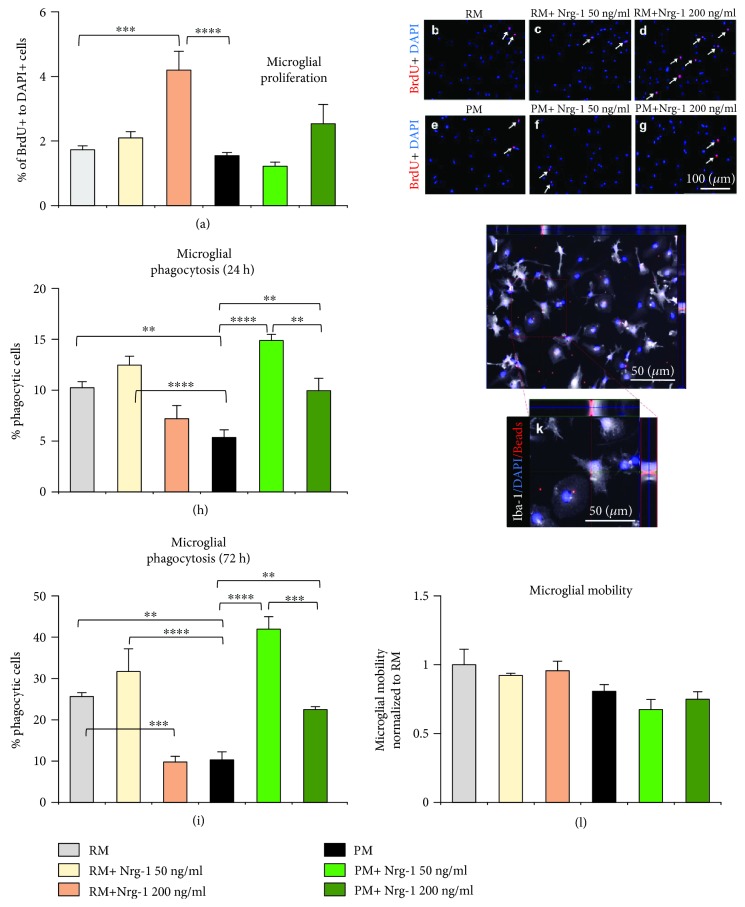
Nrg-1 treatment enhances the reduced phagocytic ability of proinflammatory microglia with no effects on their proliferation and mobility. (a-g) Immunocytochemical analysis of BrdU in microglial culture (marking proliferating cells) after 24 h exposure to Nrg-1 200 ng/ml showed a significant increase (2-folds) in proliferation of resting microglia. However, Nrg-1 treatment had no significant effects on proliferation of proinflammatory microglia at any examined concentration. White arrows indicate proliferating microglia. (h, i) For microglial phagocytosis, immunocytochemical analysis of Iba-1+/DAPI+ microglia containing red fluorescent beads indicated a significant reduction in phagocytic ability of proinflammatory microglia at 24 h and 72 h after activation. Cotreatment of proinflammatory microglia with Nrg-1 (50 and 200 ng/ml) significantly restored the percentage of phagocytic proinflammatory microglia. (j, k) To verify the occurrence of phagocytosis, we captured *Z*-stack imaging of microglia and confirmed the presence of intracellular red fluorescent beads in Iba-1+/DAPI+ microglia. (l) For microglial mobility, our quantitative analysis of DAPI+ microglia mobilized to the bottom chamber of Transwell culture inserts indicated no apparent difference in the mobility of resting and proinflammatory microglial cultures with and without Nrg-1 treatment. The data represent mean ± SEM, ^∗∗^ *p* < 0.01, ^∗∗∗^ *p* < 0.001, and ^∗∗∗∗^ *p* < 0.0001, *N* = 3 − 4 independent cultures, one-way ANOVA, followed by the Holm–Sidak post hoc test.

Following injury to the CNS, activated microglia proliferate and migrate towards the injury site and contribute to neuroinflammation [46, 47]. Our findings show that Nrg-1 200 ng/ml significantly increased proliferation of resting microglia, while there was no effect on proinflammatory microglia (Figures 3(a)–3(g)). Our quantitative immunocytochemical analysis of microglial proliferation using BrdU assay revealed a significant 2.4-fold increase in the percentage of proliferating resting microglia (BrdU+/DAPI+ cells) under Nrg-1 (200 ng/ml) treatment compared to nontreated resting microglia (*p* < 0.001, one-way ANOVA, *N* = 4 independent cultures). It is noteworthy that the Nrg-1-induced increase in microglial proliferation was observed in 4.3% of resting microglia as compared to 1.7% of baseline proliferation in these cultures indicating a low proliferative activity in these cells in serum-free conditions that we used in our studies. At 24 h following activation, there was no difference in the number of BrdU+/DAPI+ microglia between resting and activated conditions. We also found that Nrg-1 treatment (50 and 200 ng/ml) had no effect on microglial proliferation in proinflammatory cultures.

In CNS inflammatory conditions, IFN-*γ* and TNF-*α* induce a proinflammatory phenotype in microglia that is associated with their reduced phagocytic activity [21]. In contrast, anti-inflammatory microglia show increased ability for phagocytosis of cellular debris which promotes wound healing and tissue repair [13, 48]. Here, we examined whether Nrg-1 can promote the phagocytic activity of proinflammatory microglia. Phagocytosis was assessed in cultures of resting and proinflammatory microglia using serum-preopsonized red fluorescent beads. The extent of phagocytosis was determined by quantifying the number of Iba-1+/DAPI+ cells containing beads normalized to the total number of Iba-1+/DAPI+ microglia. Our quantitative analysis of phagocytosis in proinflammatory microglia at 24 h after activation indicated a significant 48% reduction in their phagocytosis. Interestingly, Nrg-1 50 ng/ml (2.7 folds) and Nrg-1 200 ng/ml (1.8 folds) significantly restored the reduced ability of proinflammatory microglia for phagocytosis (*p* < 0.01 and *p* < 0.0001, one-way ANOVA, *N* = 4 independent cultures) (Figure 3(h)). Likewise, proinflammatory microglia activated for 72 h showed a significantly diminished phagocytic activity by 60% compared to their nonactivated resting counterparts which was significantly restored with Nrg-1 50 ng/ml (4 folds) and 200 ng/ml (2.1 folds) (*p* < 0.01 and *p* < 0.0001, one-way ANOVA, *N* = 4 independent cultures) (Figure 3(i)). Our studies showed no apparent effect of Nrg-1 50 ng/ml treatment on the ability of resting microglia for phagocytosis. However, there was a significant reduction in phagocytosis in Nrg-1 200 ng/ml-treated resting microglia cultures at the 72 h time point (*p* < 0.001, one-way ANOVA, *N* = 4 independent cultures) (Figure 3(i)). Of note, we verified the specificity of phagocytosis through the detection of intracellular red fluorescent beads in Iba-1+/DAPI+ microglia using *Z*-stack imaging (Figures 3(j) and 3(k)).

We next investigated whether Nrg-1 treatment plays a role in microglial mobility using Transwell cell mobility assay. Our quantification of the total number of DAPI+ microglia which had mobilized through Transwell showed no apparent difference between the mobility of resting and proinflammatory microglia. We also found no change in microglial mobilization under Nrg-1 treatment in resting or proinflammatory cultures (*N* = 3 independent cultures) (Figure 3(l)).

### 3.4. Nrg-1 Restores the Reduced Proliferation of NPCs by Proinflammatory Microglia Partially through Downregulation of TNF-*α*

We next determined whether Nrg-1 modulation of microglia may influence the regenerative properties of adult NPCs including their capacity for proliferation, differentiation, and mobilization. Three days following microglial activation and Nrg-1 treatment, MCM was collected and added to NPC cultures. First, we verified that proinflammatory MCM did not contain any residual of the original IFN-*γ* and TNF-*α* recombinant peptides that we used for microglial activation. This analysis was performed to rule out the potential direct effects of exogenous IFN-*γ* and TNF-*α* recombinant peptides in MCM on NPC activities. We conducted Western blotting on MCM at the 24 h and 72 h time points for IFN-*γ* and TNF-*α* (Supplementary Figures 3a and 3b). Our Western blot analysis unraveled that IFN-*γ* and TNF-*α* peptides were not present in PMCM. The specificity of our Western blot analysis for detection of IFN-*γ* and TNF-*α* was verified using positive and negative controls and recombinant IFN-*γ* and TNF-*α* peptides. As expected, our results showed the endogenous expression of the pro and active forms of TNF-*α* protein in PMCM as compared to undetectable levels of both TNF-*α* isoforms in RMCM (Supplementary Figure 3a). Conversely, we found no detectable expression of IFN-*γ* cytokine in resting and proinflammatory MCM, while there were considerable levels of IFN-*γ* in the spinal cord tissue samples of experimental autoimmune encephalomyelitis (EAE) mice as positive controls (Supplementary Figure 3b). Taken together, MCM that was used in our NPC studies did not contain any detectable residual of recombinant IFN-*γ* and TNF-*α* peptides as verified by Western blotting.

**Figure 4:**
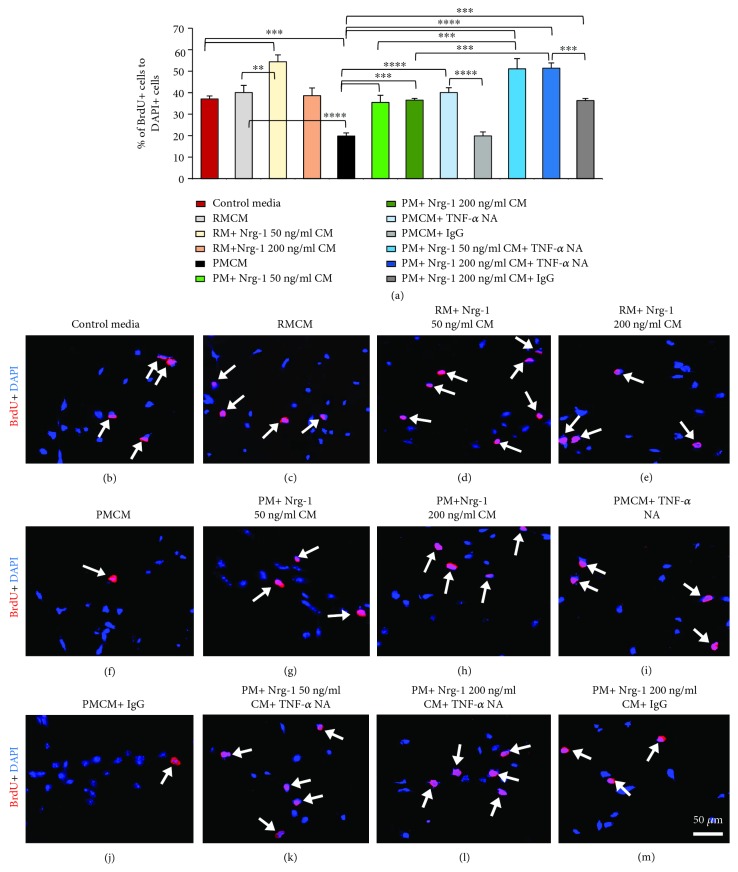
Proinflammatory microglia inhibit NPC proliferation, and Nrg-1 treatment reverses this inhibition seemingly through reduction in TNF-*α* expression. (a-m) BrdU proliferation assay showed that Nrg-1-treated resting microglia increase NPC proliferation as compared to control media and RMCM. Exposure of NPCs to PMCM significantly decreased the proliferative activity of NPCs. Nrg-1 treatment at both 50 and 200 ng/ml was able to reverse the proinflammatory-induced inhibition of NPC proliferation. When TNF-*α* was neutralized in proinflammatory MCM by TNF-*α* neutralizing Ab (NA), the inhibitory effects of proinflammatory microglia on NPC proliferation was recovered to the same level as control media and RMCM conditions suggesting this effect was mediated through TNF-*α* expression. Addition of TNF-*α* NA to Nrg-1 treated PMCM further additively promoted NPC proliferation. White arrows indicate proliferating NPCs. The data represent mean ± SEM, ^∗∗^ *p* < 0.01, ^∗∗∗^ *p* < 0.001, and ^∗∗∗∗^ *p* < 0.0001, *N* = 3 independent cultures, one-way ANOVA, followed by Holm–Sidak *post hoc* test.

For assessment of NPC proliferation, we added MCM to NPC cultures. We utilized defined NPC serum-free media (SFM) in these experiments. Dissociated NPCs were treated with the conditioned media collected from the following conditions: (1) RM, (2) RM+Nrg-1 50 ng/ml, (3) RM+Nrg-1 200 ng/ml, (4) PM, (5) PM+Nrg-1 50 ng/ml, and (6) PM+Nrg-1 200 ng/ml. NPCs were plated in control media which served as the baseline control for quantitative analysis of NPC proliferation. Our BrdU proliferation assays showed the ability of Nrg-1 treated microglia to increase NPC proliferation (Figures 4(a)–4(m)). RMCM treated with Nrg-1 50 ng/ml induced a significant 1.4-fold and 1.3-fold increase in the percentage of proliferating NPCs, marked as BrdU+/DAPI+, compared to NPCs treated with control media and RMCM (*p* < 0.01 and *p* < 0.001, one-way ANOVA, *N* = 3 independent cultures). Interestingly, PMCM resulted in a significant 46.5% and 50.5% reduction in the percentage of proliferating NPCs as compared to control media and RMCM conditions, respectively (*p* < 0.001 and *p* < 0.0001, one-way ANOVA, *N* = 3 independent cultures). Importantly, proinflammatory microglia treated with Nrg-1 (50 and 200 ng/ml) had no inhibitory effects on NPC proliferation. Our quantitative analysis of proliferating BrdU+NPCs showed that MCM collected from Nrg-1-treated proinflammatory microglia was not inhibitory and entirely recovered the proinflammatory-induced reduction in NPC proliferative activity to a comparable level as control media and RMCM (*p* < 0.001, one-way ANOVA, *N* = 3 independent cultures).

To identify a plausible mechanism by which Nrg-1 treatment reduces the inhibitory effects of proinflammatory microglia on NPC proliferation, we next asked whether Nrg-1 exerts its effects by modulating TNF-*α* expression in proinflammatory microglia. Previous studies showed an inhibitory role of TNF-*α* in NPC proliferation [49, 50]. Our cytokine expression data in this study also showed the ability of Nrg-1 to reduce TNF-*α* expression in proinflammatory microglia. To address this question, we preincubated PMCM with and without Nrg-1 (50 and 200 ng/ml) with a TNF-*α*-neutralizing antibody (NA). Our analysis showed that treating NPC cultures with PMCM preincubated with TNF-*α* NA was able to entirely reverse the inhibitory effects of PMCM on NPC proliferation and bring it to a comparable level as NPCs treated with control media or RMCM (*p* < 0.0001, one-way ANOVA, *N* = 3 independent cultures) (Figures 4(a)–4(m)). Interestingly, treatment with TNF-*α* NA additionally augmented the positive effects of Nrg-1 on NPC proliferation as there was a significant 2.5- and 1.4-fold increase in the number of proliferating cells in PM+Nrg-1 CM+TNF-*α* NA conditions as compared to cultures treated only with PM and PM+Nrg-1 CM, respectively (*p* < 0.001 and *p* < 0.0001, one-way ANOVA, *N* = 3 independent cultures) (Figures 4(a)–4(m)). Of note, we confirmed the specificity of TNF-*α* NA function using normal mouse IgG as a neutral control antibody (Figures 4(j) and 4(m)). Treating NPCs with preincubated PMCM or PM+Nrg-1 200 ng/ml CM with IgG was not able to reverse the inhibitory effects of PMCM or augment the promoting effects of Nrg-1 on NPC proliferation. These data collectively show that TNF-*α* plays an important contribution to proinflammatory-mediated inhibition of NPC proliferation and activation. Importantly, we show that availability of Nrg-1 in the microenvironment of proinflammatory microglia harnesses their ability to promote NPC proliferation by reducing TNF-*α* release.

In these experiments, we confirmed that the effects of PMCM and Nrg-1-treated MCM on NPC proliferation were not due to the presence of IFN-*γ*, TNF-*α*, and rhNrg-1 peptide per se. It was important to rule out this possibility because IFN-*γ*, TNF-*α*, and Nrg-1 are known for their direct effects on NPC proliferation [26, 50, 51]. To this end, we treated NPCs with fresh and 72 h incubated IFN-*γ* (40 ng/ml)+TNF-*α* (50 ng/ml) combination and Nrg-1 (50 and 200 ng/ml). Our verification studies, as anticipated, confirmed that direct addition of fresh Nrg-1 (50 and 200 ng/ml) significantly increased the percentage of proliferating BrdU+NPCs as compared to control media (*p* < 0.0001, one-way ANOVA, *N* = 4, independent cultures) (Supplementary Figures 4a-4h). In contrast, cotreating NPCs with fresh IFN-*γ* and TNF-*α* significantly reduced NPC proliferation. Next, to test whether residual IFN-*γ*, TNF-*α*, and rhNrg-1 peptide in MCM had any direct effects on NPCs, we incubated the peptides for 72 h prior to its addition to NPC cultures. This mimicked the duration of our treatments in microglial culture. Our quantitative analysis showed that treating NPCs directly with 72 h incubated IFN-*γ*+TNF-*α* combination or Nrg-1 (50 and 200 ng/ml) had no apparent effects on NPCs' proliferative activity (*p* > 0.05, one-way ANOVA, *N* = 4, independent cultures). These verification studies confirmed that the effects of the CM, collected from microglia, on NPC cultures were attributed to the IFN-*γ*+TNF-*α*- or Nrg-1-modulated microglial secretion and not the original peptides *per se* (Supplementary Figures 4a–4h).

### 3.5. Proinflammatory Microglia Suppress NPC Differentiation through TNF-*α* Release

**Figure 5:**
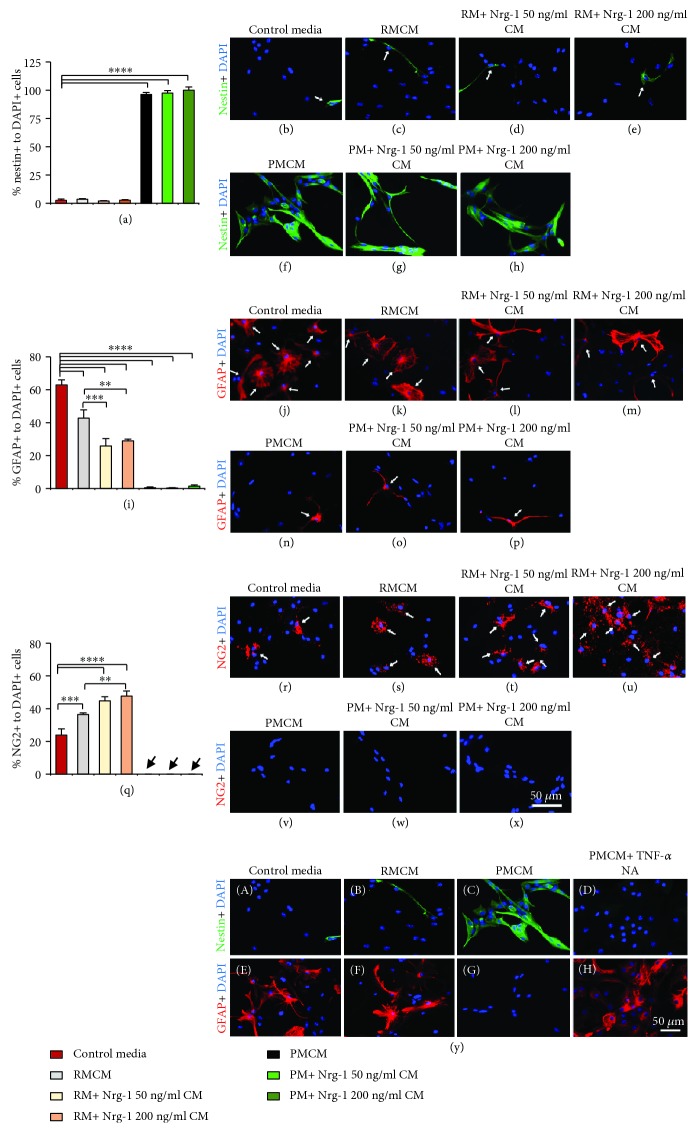
Proinflammatory microglia block the differentiation of adult NPCs through TNF-*α* release. Immunocytochemistry was performed to assess NPC differentiation using an NPC-specific marker, nestin, the astrocyte-specific marker, GFAP, and the OPC-specific marker, NG2. (a-h) Our nestin immunocytochemistry indicated that NPCs differentiated in control media, RMCM, and RM+Nrg-1 CM conditions as nestin expression was not detected in these cultures. Conversely, nearly all NPCs under PM and PM+ Nrg-1 CM treatment remained nestin+undifferentiated cells. (i-x) Control media and RMCM induced astrocyte differentiation in NPCs (GFAP+ cells), while reducing oligodendrocyte differentiation (NG2+ cells). Interestingly, Nrg-1 (50 and 200 ng/ml) treated RMCM significantly reduced differentiation of NPCs to GFAP+/DAPI+ astrocytes and increased the percentage of NG2+/DAPI+ OPCs as compared to RMCM and control media. PMCM entirely blocked NPC differentiation to oligodendrocytes with only a small number of astrocytes. (y, A-H) Treatment of PMCM with TNF-*α* NA reversed the inhibitory effects of PMCM on NPC differentiation suggesting this effect was mediated through TNF-*α* expression by proinflammatory microglia. Black arrows in graph (q) show PMCM with and without Nrg-1 (50 and 200 ng/ml). The data represent mean ± SEM, ^∗∗^ *p* < 0.01, ^∗∗∗^ *p* < 0.001, and ^∗∗∗∗^ *p* < 0.0001, *N* = 4 independent cultures, one-way ANOVA, followed by Holm–Sidak *post hoc* test.

Next, we investigated whether resting and proinflammatory MCM with or without Nrg-1 treatment influence NPC differentiation. To allow differentiation, NPCs were grown in a medium composed of NPC SFM and microglial serum medium (1 : 1 ratio) for 7 days. We treated NPCs with RM, RM+Nrg-1 50 ng/ml, RM + Nrg-1 200 ng/ml, PM, PM + Nrg-1 50 ng/ml, and PM + Nrg-1 200 ng/ml CM. We also preincubated PMCM with TNF-*α* NA to assess the overall effects of microglia-derived TNF-*α* on NPC differentiation. NPCs were plated in control media which served as the baseline condition for quantitative analysis of differentiation. We performed immunocytochemical analysis using nestin (NPC lineage marker), GFAP (astrocyte-specific marker), and NG2 (oligodendrocyte progenitor-specific marker) (Figures 5(a)–5(y)). After 7 days of differentiation, our quantification for nestin+/DAPI+ cells showed that the majority of NPCs differentiated in the control media and RMCM with and without Nrg-1 because we only found a rather low number of nestin-positive NPCs. Of note, nestin is a signature marker for undifferentiated multipotent NPCs that are downregulated in differentiated neural lineages. On the other hand, PMCM suppressed NPC differentiation entirely because all NPCs expressed nestin (Figures 5(a)–5(h)). Treatment of proinflammatory microglia with Nrg-1 (50 and 200 ng/ml) did not change their inhibitory effects on NPC differentiation.

We next studied the differentiation pattern of NPCs. In agreement with our previous data, under control media condition, 62% of NPCs differentiated into astrocytes while 23% were NG2-expressing oligodendrocyte precursor cells (OPCs) (Figures 5(i)–5(x)). Interestingly, RMCM promoted a significant increase in the percentage of NG2+/DAPI+ OPCs while decreasing GFAP+/DAPI+ astrocytes as compared to control media (*p* < 0.001 and *p* < 0.0001 one-way ANOVA, *N* = 4 independent cultures). Nrg-1 (50 and 200 ng/ml) treatment further promoted the positive effects of RMCM in increasing the number of OPCs while reducing astrocyte differentiation. In these experiments, to rule out the possibility that the effects of Nrg-1-treated RMCM on NPC differentiation were not due to Nrg-1 peptide per se, we also treated NPCs with fresh and 72 h-incubated Nrg-1 (50 and 200 ng/ml) (Supplementary Figures 4i–4t). Previous studies by our group showed that Nrg-1 promotes NPC differentiation into oligodendrocytes at the expense of astrocyte differentiation [26]. Here, we confirmed that fresh Nrg-1 (50 and 200 ng/ml) significantly increased the percentage of NG2-positive OPCs and reduced the percentage of GFAP-positive astrocytes as compared to control media (*p* < 0.0001, one-way ANOVA, *N* = 4, independent cultures). We also tested Nrg-1 incubated for 72 h prior to its addition to NPC cultures to determine the effects and potency of any residual Nrg-1 treatment in MCM. Our quantitative analysis showed that treating NPCs directly with 72 h-preincubated Nrg-1 (50 and 200 ng/ml) had no apparent effects on NPC differentiation into OPCs (*p* > 0.05, one-way ANOVA, *N* = 4, independent cultures). This evidence confirmed that the effect of MCM that we observed in NPC cultures was due to the Nrg-1-modulated microglial secretion and not the original Nrg-1 treatment *per se* (Supplementary Figures 4i–4t).

In contrast to resting microglia, NPCs exposed to PMCM remained largely undifferentiated with no detectable oligodendrocyte differentiation and only a rare number of GFAP-positive astrocytes. Nrg-1 treatment of proinflammatory microglia was not able to reverse their inhibition on NPC differentiation. However, neutralizing TNF-*α* in PMCM completely reversed the inhibitory effects of PMCM on NPC differentiation evident by GFAP expression in NPC cultures. This observation indicated that the suppression of NPC differentiation into OPCs and astrocytes was mediated via TNF-*α* release in proinflammatory microglial cultures (Figure 5(y), (A-H)).

### 3.6. Nrg-1 Enhances the Capacity of Microglia in Supporting NPC Mobilization

**Figure 6:**
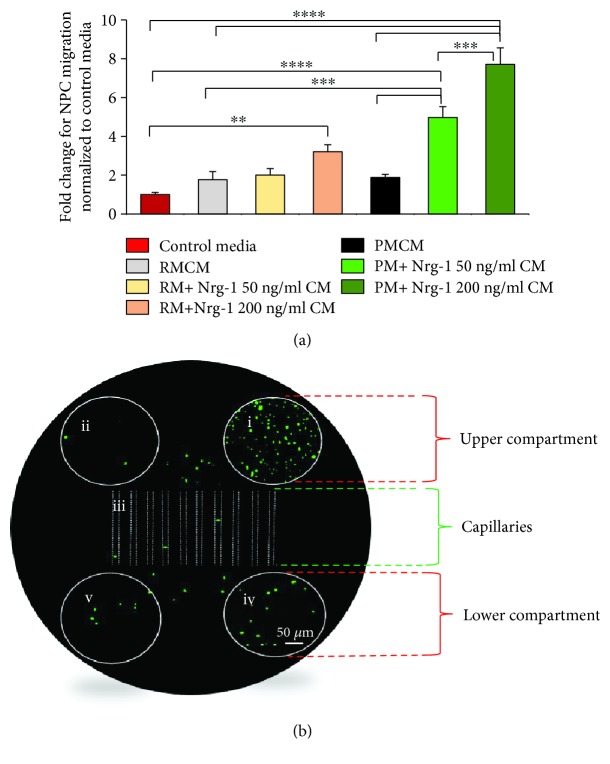
Nrg-1 augments the ability of microglia in promoting NPC mobilization. (a, b) MCM from resting microglia treated with Nrg-1 200 ng/ml significantly increased the number of mobilized NPCs. Importantly, addition of Nrg-1- (50 and 200 ng/ml) treated PMCM significantly induced mobilization of NPCs in a dose-dependent manner compared to all other conditions. (b, i-v) The majority of cells move from the right-sided well in the upper compartment (lower gradient slope) toward the lower compartment (higher gradient slope) through capillaries. NPCs passing through capillaries can enter the wells or the area between two wells in the lower compartment. NPCs moving into the capillaries and lower compartment were counted as mobilized cells for each experimental condition. A few NPCs migrate from the right-sided well to the left-sided well in the upper compartment of each Ananda microdevice. The data represent mean ± SEM, ^∗∗^ *p* < 0.01, ^∗∗∗^ *p* < 0.001, and ^∗∗∗∗^ *p* < 0.0001, *N* = 3 independent cultures, one-way ANOVA, followed by Holm–Sidak *post hoc* test.

In the CNS, microglia are shown to influence NPC mobilization and regulate the ability of NPCs to respond to injury [52]. Here, we examined whether presence of Nrg-1 in a microglial environment has any effects on their influence on NPC mobilization. Three days following microglial activation, CM was collected from the following conditions: (1) RM, (2) RM+Nrg-1 50 ng/ml, (3) RM+Nrg-1 200 ng/ml, (4) PM, (5) PM+Nrg-1 50 ng/ml, and (6) PM+Nrg-1 200 ng/ml. MCM was added to the lower compartment of each Ananda microdevice for assessment of NPC mobilization (Figures 6(a) and 6(b)). Dissociated NPCs were plated onto the right-sided well in the upper compartment of the microdevice, and cells were allowed to migrate from the upper compartment through capillaries toward the lower compartment for 20 h (Figure 6(b), i-v). Our quantification showed that MCM of Nrg-1-treated microglia stimulates mobilization of NPCs in a concentration-dependent manner. MCM of Nrg-1 200 ng/ml-treated resting microglia induced a significant 3.2-fold increase in the proportion of NPCs mobilized toward lower compartments compared to control media (*p* < 0.01, one-way ANOVA, *N* = 3 independent cultures). We also found that the MCM of Nrg-1- (50 and 200 ng/ml) treated proinflammatory microglia resulted in a significant increase in the number of mobilized NPCs as compared to control media, RMCM, and PMCM conditions (*p* < 0.001 and *p* < 0.0001, one-way ANOVA, *N* = 3 independent cultures). Our quantitative analysis showed a significant 1.5-fold increase in the number of mobilized NPCs in the PM+200 ng/ml CM condition as compared to the PM+Nrg-1 50 ng/ml CM condition (*p* < 0.001, one-way ANOVA, *N* = 3 independent cultures).

Similar to our NPC proliferation and differentiation studies, we ruled out the possibility that the residual of the Nrg-1 recombinant peptide in MCM may affect NPC mobilization. To confirm this, we added fresh and 72 h preincubated Nrg-1 (50 and 200 ng/ml) to wells in the lower compartment of microdevices (Supplementary Figures 5a and 5b, i-v). We found that fresh Nrg-1 at 200 ng/ml significantly induced the proportion of mobilized NPCs as compared to control media (*p* < 0.001, one-way ANOVA, *N* = 3 independent cultures). However, there was no difference in NPC mobilization between 72 h-incubated Nrg-1 (at all examined concentrations) and control media (*p* > 0.05, *N* = 3 independent cultures). This evidence confirmed that the effect of MCM that we observed in NPC cultures was due to the Nrg-1-modulated microglial secretion and not the original Nrg-1 treatment per se.

## 4. Discussion

In the present study, we demonstrate, for the first time, a beneficial role for Nrg-1 in regulating microglial response and their influence on the regenerative properties of adult NPCs. Using primary culture systems and an injury-relevant activation strategy, we demonstrate that availability of Nrg-1 in the microenvironment of proinflammatory microglia moderates their phenotype and harnesses their potential to promote repair process. We found that Nrg-1 is able to attenuate the expression of key proinflammatory cytokines TNF-*α* and IL-6 in proinflammatory microglia. Importantly, we have provided novel evidence that Nrg-1 availability can restore the otherwise reduced ability of proinflammatory microglia for phagocytosis, an essential step in wound healing and tissue repair in CNS injury. Moreover, our new findings indicate that the modulatory effects of Nrg-1 on proinflammatory microglia can promote their interactions with NPCs and increase their ability to support proliferation and mobilization of NPCs. Altogether, our findings suggest a positive regulatory role for Nrg-1 in microglia/NPC crosstalk that can be exploited to facilitate the cellular repair process in CNS injury and disease.

NPCs have an innate potential for neural repair and regeneration. However, in the injured CNS, resident and transplanted NPCs show restricted regenerative capacity to replenish damaged neurons and oligodendrocytes [53, 54]. This limitation has been attributed to the hostile milieu of the injured CNS partly driven by the glial scar and proinflammatory response of immune cells and activated microglia [54, 55]. In the developing and adult CNS, microglia and their secreted factors support NPC survival, proliferation, mobilization, and differentiation [56–58]. The absence of microglia during embryogenesis results in decreased NPC proliferation and differentiation [59]. Evidence shows that microglia exert regulatory effects on hippocampal neurogenesis by pruning newborn neurons and removing apoptotic cells without inducing inflammation [5, 60, 61]. Microglia-secreted factors also promote the survival and maturation of neurons [62–64]. Altogether, it is well-established that microglia support NPCs in their healthy CNS niche. However, in the injured or diseased CNS, activated microglia play differently and can contribute to both tissue damage and repair owing to the diverse phenotypes that they acquire during the inflammatory process. In the pathologic CNS, microglial function largely reflects their activation state and the balance between their proinflammatory and anti-inflammatory/proregenerative phenotypes [65]. Microenvironmental changes associated with CNS injury determine microglial phenotype and consequently their effects on NPCs [66]. We and others have shown that proinflammatory and proregenerative microglia differentially regulate the response of NPCs following CNS injuries or diseases [21, 49, 67, 68]. Proregenerative microglia promote oligodendrocyte differentiation and remyelination through the secretion of IGF-I and TGF-*β*1 [69, 70]. Exposure of microglia to IL-4 can favor the fate of NPCs towards oligodendrogenesis or neurogenesis through IGF-I expression. Conversely, LPS-induced proinflammatory microglia block NPC differentiation via TNF-*α* expression [17]. Therefore, understanding how microglia are regulated in their environment would aid in identifying new immunomodulatory strategies that can harness their potential for CNS repair.

We previously demonstrated that downregulation of Nrg-1 within the lesions of SCI and focal demyelinating conditions contributes to the imbalanced microenvironment of injury and underlies the inadequate oligodendrocyte differentiation by endogenous precursor cells [25, 26]. Our parallel studies identified that exogenous Nrg-1 can enhance the capacity of spinal cord precursor cells for proliferation and oligodendrocyte differentiation *in vitro* and in the injured spinal cord [25, 26]. Notably, our recent work in SCI identified a positive role for Nrg-1 in regulating neuroinflammation [23, 24]. Microglia are known to be responsive to Nrg-1 bioavailability through the expression of ErbB 2, 3, and 4 receptors [23, 38]. These findings collectively suggest that Nrg-1 can promote the response of endogenous precursor cells through direct regulation of NPCs and potentially indirectly by modulating the inflammatory response including microglial activity.

In this study, we provide new evidence that Nrg-1 can promote the reparative properties of NPC indirectly by forging a supportive phenotype in proinflammatory microglia. Proinflammatory microglia are known to promote tissue degeneration and hinder the repair process through their proinflammatory properties and their reduced efficacy in clearance of myelin debris [16, 71–74]. Here, we demonstrate that Nrg-1 directly affects mouse proinflammatory microglia in culture and mitigates their levels of proinflammatory markers, NO, TNF-*α*, IL-6, and CD86. Of note, we conducted several assessments to confirm that NPCs are specifically modulated through the factors released by microglia and not by the residual recombinant peptides, IFN-*γ*, TNF-*α*, and Nrg-1, which were used exogenously in our experiments. Interestingly, in our previous studies, Nrg-1 treatment remarkably increased the protein levels of IL-10 in anti-inflammatory microglia/macrophages as well as T and B regulatory cells in SCI and focal demyelinating lesions of the spinal cord [23, 25]. However, in this study, Nrg-1 treatment was not able to significantly restore the reduced expression of IL-10 in proinflammatory microglia *in vitro*. This observation suggests that the increase in IL-10 and Arg-1 expression after Nrg-1 treatment in SCI may be primarily attributed to monocyte-derived macrophages as well as T and B regulatory cells that infiltrate the injured spinal cord. However, it is plausible that Nrg-1 may affect other anti-inflammatory/proregenerative mediators expressed by microglia that were not studied in our experiments. Further large-scale phenotypic analysis is required to fully understand the modulatory effects of Nrg-1 on microglia.

Recruitment of NPCs is critical for endogenous repair after CNS injury and diseases [75, 76]. Here, we demonstrate that Nrg-1 treatment fosters a supportive phenotype in proinflammatory microglia, which promotes proliferation and mobilization of NPCs. Evidence from our present study and that of others show that proinflammatory microglia restrict the regenerative properties of NPCs [77]. Here, under TNF-*α* and IFN-*γ* treatment, mouse proinflammatory microglia significantly repressed the proliferative activity of adult NPCs. This inhibition was entirely reversed by the availability of Nrg-1 in the environment of proinflammatory microglia. Additionally, we found that Nrg-1-treated resting microglia stimulated the proliferative ability of NPCs suggesting a supportive role for Nrg-1 in regulating microglia under homeostasis condition. Mechanistically, we provide new evidence that Nrg-1 restores the repressive effects of proinflammatory microglia on NPC activation partially by mitigating the production of TNF-*α*. Previous studies have also identified a suppressive role for TNF-*α* in regulating NPCs in the SVZ and hippocampus [49, 78]. These studies showed that TNF-*α* reduces NPC proliferation in the brain through receptor-mediated mechanisms in both normal and injury conditions. In our experiments, while Nrg-1 treatment or TNF-*α* neutralization was able to overcome proinflammatory-mediated repression of NPC proliferation individually, in combination they additively promoted a higher degree of proliferation in NPCs. These observations indicate that Nrg-1 appears to modulate microglial regulation of NPC proliferation through TNF-*α* independent mechanisms as well. This interpretation was indeed corroborated when Nrg-1 treatment also augmented the ability of resting microglia in promoting NPC proliferation under homeostasis conditions in which TNF-*α* is not normally produced. We also show that the MCM of Nrg-1-modulated microglia have the capacity to induce NPCs mobilization in a dose-dependent manner. Mobilization of NPCs is an important aspect of their behavior. In the normal CNS, NPCs migrate from the SVZ to the olfactory bulbs along the rostral migratory stream (RMS) to replace olfactory neurons [79, 80]. After CNS injury or disease, mobilization of resident NPCs toward the injury site is critical for the repair process [81–83]. Several inhibitory and promoting factors have been identified that regulate mobilization of precursor cells following CNS injury or disease including microglial secretory factors [52]. Future investigations are needed to unravel the mechanisms by which Nrg-1 modulates microglia/NPC crosstalk.

In the injured or diseased CNS, differentiation of NPCs is also restricted by proinflammatory glial and immune cells [55]. Our findings show that exposure of NPCs to proinflammatory microglia-conditioned media entirely blocked their differentiation and neutralization of TNF-*α* in MCM was sufficient to reverse this inhibition entirely. Our findings are in agreement with previous studies that showed LPS-induced proinflammatory microglia express high levels of TNF-*α*, which blocks differentiation and cell renewal of mouse brain-derived NPCs [17]. Interestingly, we found that Nrg-1 had no effects on proinflammatory microglia-mediated suppression of NPC differentiation, while it was able to augment the ability of resting microglia in promoting differentiation of NPCs into oligodendrocytes. The inability of Nrg-1 in overcoming the proinflammatory inhibitory effects on NPC differentiation may reflect the robust effect of TNF-*α* on NPC differentiation and the fact that Nrg-1 was not able to reduce TNF-*α* expression in proinflammatory microglia to its basal level detected in resting microglia. Altogether, our studies suggest that Nrg-1 has differential roles in microglia- mediated effects on NPC proliferation and differentiation.

Another novel finding in our study is the positive effects of Nrg-1 on the phagocytic activity of proinflammatory microglia. Impaired ability of microglia for phagocytosis is an underlying cause for limited tissue regeneration after injury [84–86]. Thereby, promoting microglial phagocytosis is critical for recovery from CNS injury or disease [87, 88]. Here, for the first time, we provide evidence that Nrg-1 fosters a proregenerative phenotype in microglia by improving the suppressed phagocytic capacity of proinflammatory microglia. Our findings also show that Nrg-1 reduced phagocytosis of resting microglia particularly at a higher concentration. This observation indicates that Nrg-1 may exert differential effects on microglial phagocytosis depending on their state of activation at least under our *in vitro* conditions. Further studies are required to understand the underlying mechanism(s) by which Nrg-1 regulates microglial phagocytosis under different activation states.

## 5. Conclusions

We provide new evidence that Nrg-1 fosters a supportive phenotype in microglia under both normal and injury states, which enhances the reparative capacity of NPCs. Moreover, we have identified that bioavailability of Nrg-1 in the microenvironment of proinflammatory microglia moderates their response and augments their otherwise reduced ability for phagocytosis. Hence, we propose that Nrg-1 plays an important regulatory role in microglial function and its availability can promote the outcome of microglial response during CNS injury and repair.

## Figures and Tables

**Table 1:** List of primers used in this study.

Genes	Primer	Size
*CD86*	F: GAAGCCGAATCAGCCTAGCAG	132
	R: CACTCTGCATTTGGTTTTGCTGAAG	

*H2afz*	F: CTCACCGCAGAGGTACTTGA	95
	R: CCACGTATAGCAAGCTGCAAG	

*IL-6*	F: TCCTCTCTGCAAGAGACTTCC	96
	R: AGTCTCCTCTCCGGACTTGT	

*IL10*	F: ACTTGGGTTGCCAAGCCTTA	160
	R: AGAAATCGATGACAGCGCCT	

*TNF-a*	F: GTAGCCCACGTCGTAGCAAAC	99
	R: GTCTTTGAGATCCATGCCGTTG	

**Table 2:** List of antibodies used in this study.

Antibody	Source	Application	Dilution factor
Alexa Fluor® 568 goat anti-Chicken	Invitrogen (chicken)	ICC	1 : 600
Alexa Fluor® 568 goat anti-mouse	Invitrogen (mouse)	ICC	1 : 600
Alexa Fluor® 568 goat anti-Rabbit	Invitrogen (rabbit)	ICC	1 : 600
Alexa Fluor® 647 goat anti-mouse	Invitrogen (mouse)	ICC	1 : 600
Alexa Fluor® 647 goat anti-Rabbit	Invitrogen (rabbit)	ICC	1 : 600
BrdU	BD (mouse)	ICC	1 : 400
ErbB2	Santa Cruz (rabbit)	ICC	1 : 100
ErbB3	Santa Cruz (rabbit)	ICC	1 : 100
ErbB4	Santa Cruz (rabbit)	ICC	1 : 100
GAPDH	Santa Cruz (rabbit)	WB	1 : 1000
Beta-actin	Chemicon (mouse)	WB	1 : 500
GFAP	DAKO (rabbit)	ICC	1 : 800
Iba-1	Wako (rabbit)	ICC	1 : 500
IFN-*γ*	R&D (rat)	WB	1 : 1000
Nestin	AVES (chicken)	ICC	1 : 500
NG2	Chemicon (rabbit)	ICC	1 : 300
OX42 (CD11b)	Cedarlane (mouse)	ICC	1 : 100
TNF-*α*	Serotec (rabbit)	WB	1 : 1000
Arginase-1	Cell signaling (rabbit)	WB	1 : 1000

## Data Availability

The data used to support the findings of this study are available from the corresponding author upon request.

## Abbreviations

CNS: Central nervous system
Nrg-1: Neuregulin-1
NPCs: Neural precursor cells
NO: Nitric oxide
TNF-*α*: Tumor necrosis factor-*α*
IL-6: Interleukin-6
CD86: Cluster of differentiation 86
IL-10: Interleukin-10
MCM: Microglia-conditioned media
RM: Resting microglia
PM: Proinflammatory microglia
MS: Multiple sclerosis
OPCs: Oligodendrocyte precursor cells
EYFP Tg: Enhanced yellow fluorescent protein-tagged
aCFS: Artificial cerebrospinal fluid solution
PSN: Penicillin–streptomycin–neomycin
FBS: Fetal bovine serum
PEI: Polyethylenimine
PBS: Phosphate-buffered saline
SFM: Serum free media
IFN-*γ*: Interferon-*γ*
PFA: Paraformaldehyde
rhNrg-1*β*1: Recombinant human Nrg-1*β*1
PDL: Poly-D-lysine
SVZ: Subventricular zone
NA: Neutralizing antibody
EAE: Experimental autoimmune encephalomyelitis
Iba-1: Ionized calcium binding adaptor molecule 1
GFAP: Glial fibrillary acidic protein
NG2: Neuron glial antigen-2
DAPI: 4′,6-Diamidino-2-phenylindole
BrdU: 5-Bromo-2′-deoxyuridine
Arg-1: Arginase-1.

## Supplementary Materials

Supplementary 1 Supplementary Figure 1: confirmatory evidence that mouse microglia express all Nrg-1 signaling receptors.

Supplementary 2 Supplementary Figure 2: Nrg-1 significantly ameliorates TNF-*α* protein expression while has no effect on arginase-1 expression in proinflammatory microglia.

Supplementary 3 Supplementary Figure 3: confirmatory evidence that conditioned media collected from proinflammatory microglia (PMCM) did not contain a detectable level of the original IFN-*γ* and TNF-*α* peptides that were used for activation.

Supplementary 4 Supplementary Figure 4: confirmatory evidence to verify that the original IFN-*γ*, TNF-*α*, and Nrg-1 peptides did not affect NPC properties.

Supplementary 5 Supplementary Figure 5: evidence to verify that the original Nrg-1 treatment in MCM did not affect the outcomes of NPC mobilization.
