# Supplementary material for: Neuregulin-1 Fosters Supportive Interactions between Microglia and Neural Stem/Progenitor Cells
Source: Stem Cells Int. 2019 Apr 7;2019:8397158. doi: 10.1155/2019/8397158 (PMC6476022; doi:10.1155/2019/8397158)
Supplement: Supplementary 1 — Supplementary Figure 1: confirmatory evidence that mouse microglia express all Nrg-1 signaling receptors. [file 8397158.f1.pptx]

## Slide 1
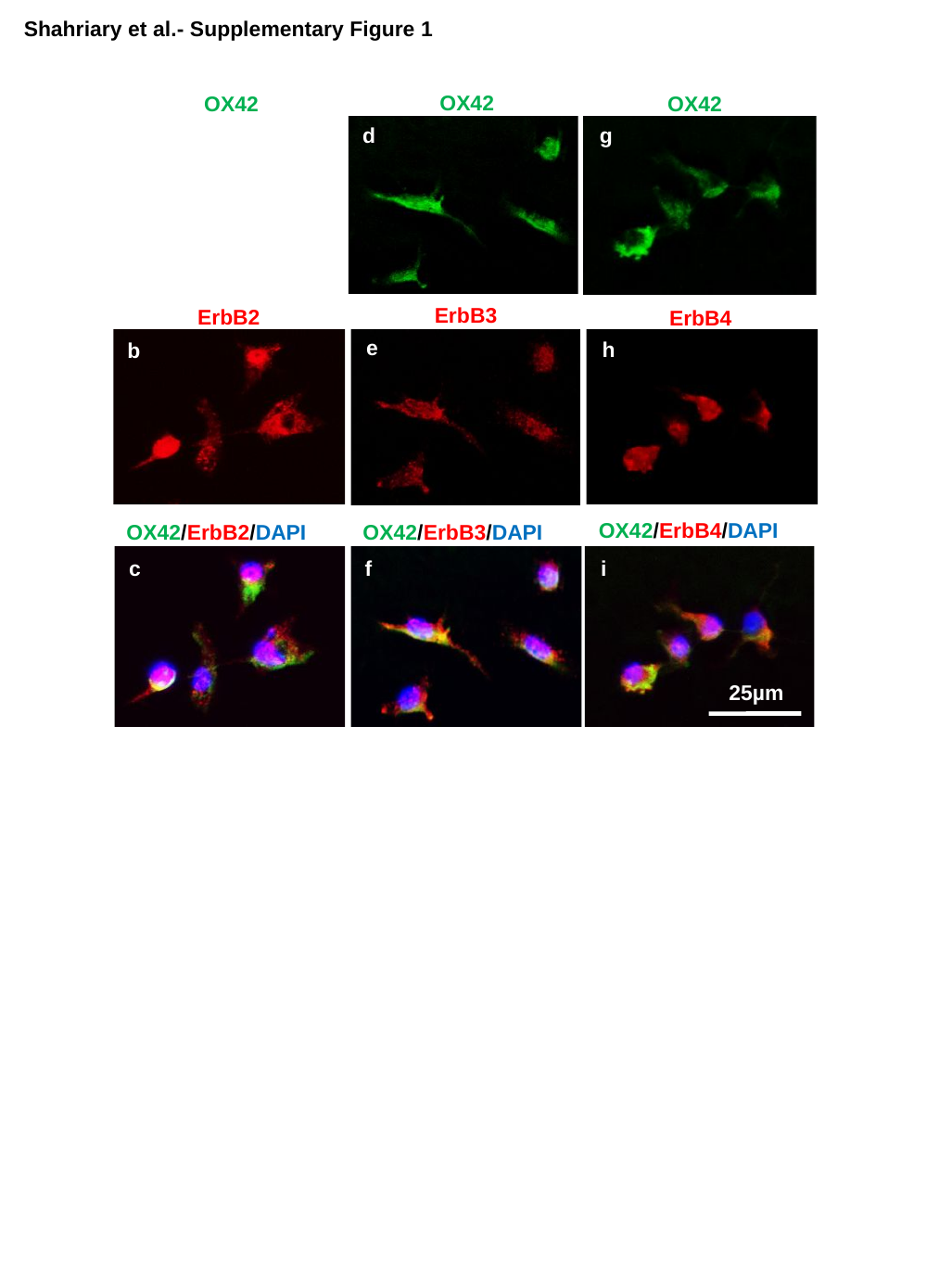

Shahriary et al.- Supplementary Figure 1
OX42
OX42
OX42
a
g
d
ErbB3
ErbB2
ErbB4
e
h
b
OX42/ErbB4/DAPI
OX42/ErbB2/DAPI
OX42/ErbB3/DAPI
f
i
c
25µm
