# Supplementary material for: Neuregulin-1 Fosters Supportive Interactions between Microglia and Neural Stem/Progenitor Cells
Source: Stem Cells Int. 2019 Apr 7;2019:8397158. doi: 10.1155/2019/8397158 (PMC6476022; doi:10.1155/2019/8397158)
Supplement: Supplementary 2 — Supplementary Figure 2: Nrg-1 significantly ameliorates TNF-α protein expression while has no effect on arginase-1 expression in proinflammatory microglia. [file 8397158.f2.pptx]

## Slide 1
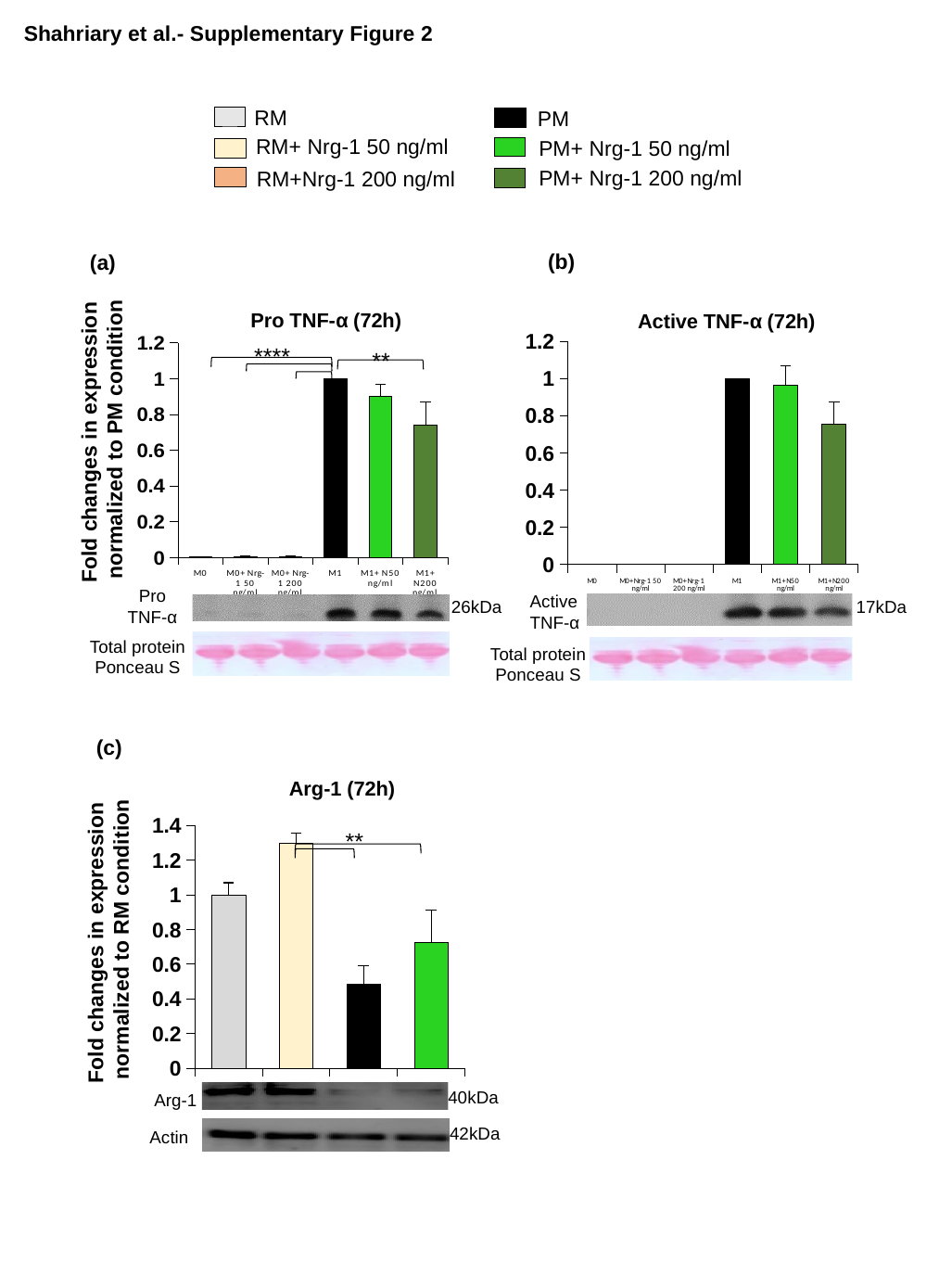

Shahriary et al.- Supplementary Figure 2
 RM
RM+ Nrg-1 50 ng/ml
RM+Nrg-1 200 ng/ml
PM
PM+ Nrg-1 50 ng/ml
PM+ Nrg-1 200 ng/ml
(b)
Active TNF-α (72h)
### Chart
| Category | |
|---|---|
| M0 | 0.0 |
| M0+ Nrg-1 50 ng/ml | 0.0 |
| M0+ Nrg-1 200 ng/ml | 0.0 |
| M1 | 1.0000000329910892 |
| M1+ N50 ng/ml | 0.9665239194646128 |
| M1+ N200 ng/ml | 0.7579118264521464 |(a)
Pro TNF-α (72h)
### Chart
| Category | |
|---|---|
| M0 | 0.0038809144256038877 |
| M0+ Nrg-1 50 ng/ml | 0.00449335568933902 |
| M0+ Nrg-1 200 ng/ml | 0.00663085699354398 |
| M1 | 0.9999999512727045 |
| M1+ N50 ng/ml | 0.899323137756116 |
| M1+ N200 ng/ml | 0.7426675437904264 |
****
**
Fold changes in expression
normalized to PM condition
Pro
TNF-α
26kDa
Total protein
Ponceau S
Active
TNF-α
17kDa
Total protein
Ponceau S
(c)
Arg-1 (72h)
### Chart
| Category | |
|---|---|
| Control | 1.0000000209544706 |
| N | 1.2965862057619837 |
| IT | 0.4854198232557465 |
| ITN | 0.7265066484925845 |
**
Fold changes in expression
normalized to RM condition
40kDa
Arg-1
42kDa
Actin
