# Supplementary material for: Neuregulin-1 Fosters Supportive Interactions between Microglia and Neural Stem/Progenitor Cells
Source: Stem Cells Int. 2019 Apr 7;2019:8397158. doi: 10.1155/2019/8397158 (PMC6476022; doi:10.1155/2019/8397158)
Supplement: Supplementary 3 — Supplementary Figure 3: confirmatory evidence that conditioned media collected from proinflammatory microglia (PMCM) did not contain a detectable level of the original IFN-γ and TNF-α peptides that were used for activation. [file 8397158.f3.pptx]

## Slide 1
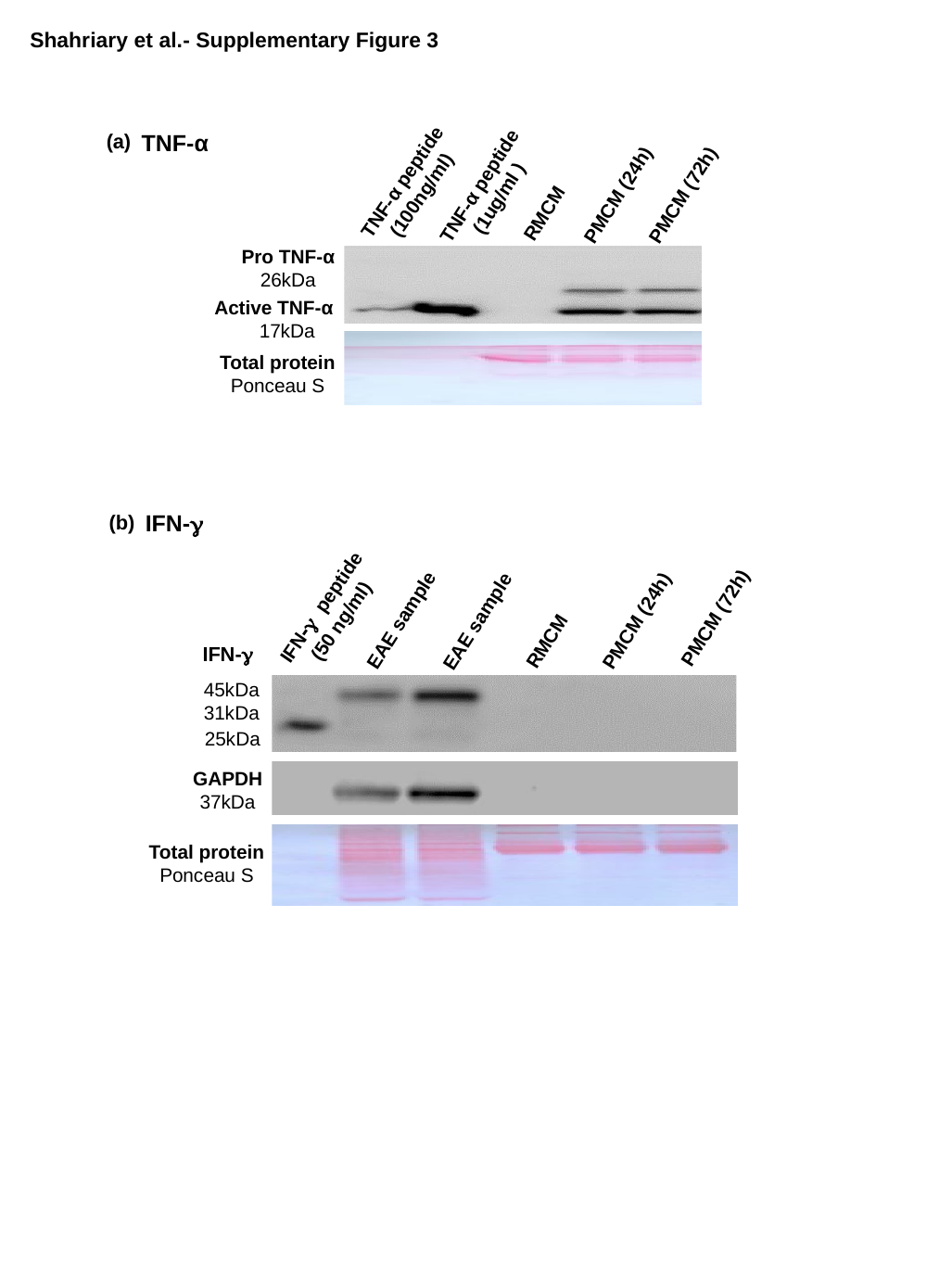

Shahriary et al.- Supplementary Figure 3
TNF-α
(a)
TNF-α peptide
(100ng/ml)
RMCM
PMCM (24h)
PMCM (72h)
Pro TNF-α
26kDa
Active TNF-α
 17kDa
Total protein
Ponceau S
TNF-α peptide
(1ug/ml )
IFN-γ peptide
(50 ng/ml)
PMCM (72h)
PMCM (24h)
RMCM
EAE sample
EAE sample
IFN-γ
45kDa
31kDa
25kDa
GAPDH
37kDa
Total protein
Ponceau S
IFN-γ
(b)
