# Supplementary material for: Neuregulin-1 Fosters Supportive Interactions between Microglia and Neural Stem/Progenitor Cells
Source: Stem Cells Int. 2019 Apr 7;2019:8397158. doi: 10.1155/2019/8397158 (PMC6476022; doi:10.1155/2019/8397158)
Supplement: Supplementary 4 — Supplementary Figure 4: confirmatory evidence to verify that the original IFN-γ, TNF-α, and Nrg-1 peptides did not affect NPC properties. [file 8397158.f4.pptx]

## Slide 1
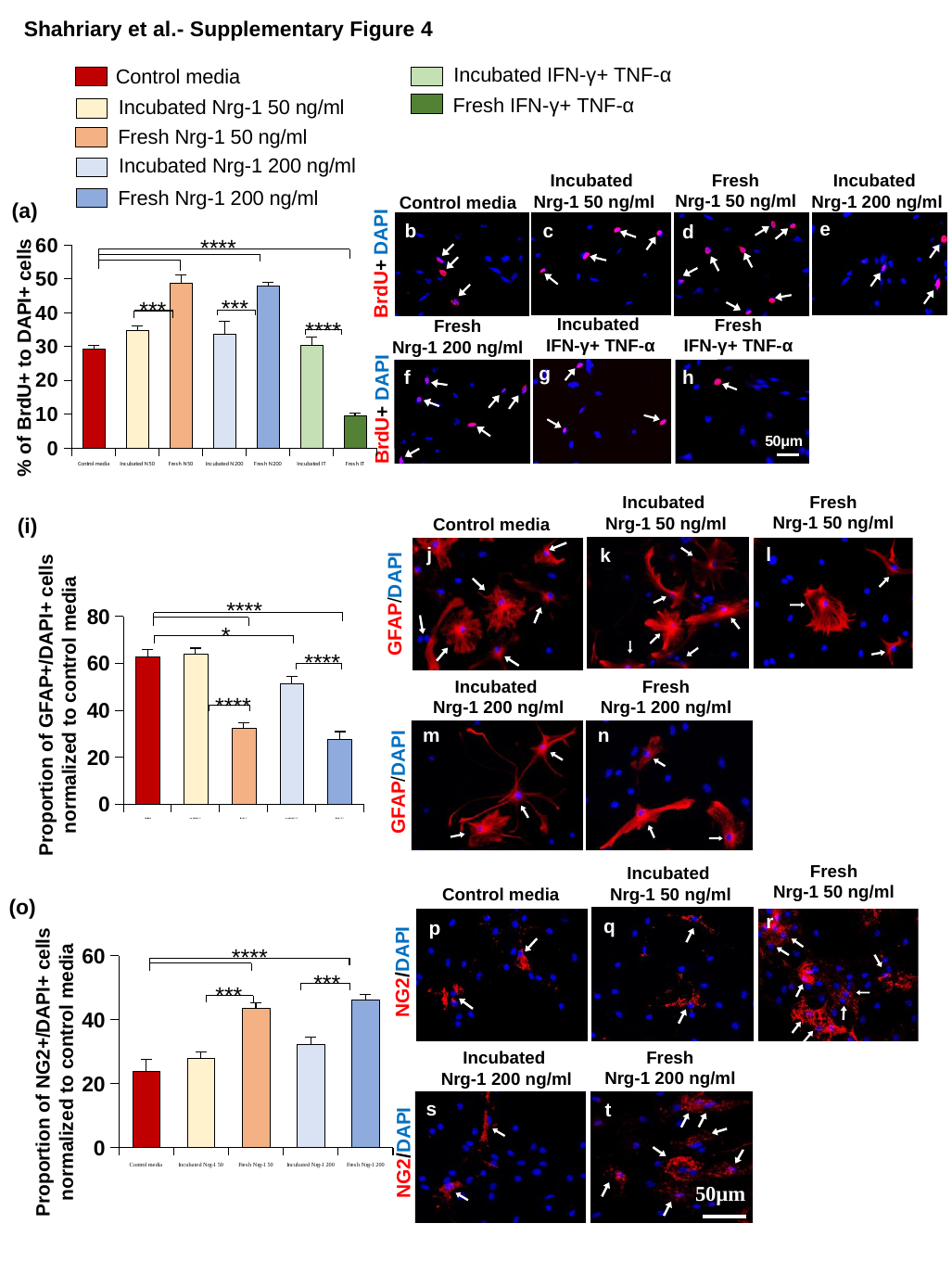

Shahriary et al.- Supplementary Figure 4
Incubated IFN-γ+ TNF-α
Control media
Fresh IFN-γ+ TNF-α
Incubated Nrg-1 50 ng/ml
Fresh Nrg-1 50 ng/ml
Incubated Nrg-1 200 ng/ml
Fresh Nrg-1 200 ng/ml
(a)
****
### Chart
| Category | |
|---|---|
| Control media | 29.39571474318509 |
| Incubated N50 | 34.76672682635489 |
| Fresh N50 | 48.79973149648683 |
| Incubated N200 | 33.559815697893924 |
| Fresh N200 | 47.80800548073283 |
| Incubated IT | 30.33281918603685 |
| Fresh IT | 9.576147566483277 |***
***
****
% of BrdU+ to DAPI+ cells
Fresh
Nrg-1 50 ng/ml
Incubated
Nrg-1 50 ng/ml
Incubated
Nrg-1 200 ng/ml
Control media
e
c
b
d
Incubated
IFN-γ+ TNF-α
Fresh
IFN-γ+ TNF-α
Fresh
Nrg-1 200 ng/ml
g
h
f
BrdU+ DAPI
BrdU+ DAPI
Fresh
Nrg-1 50 ng/ml
Incubated
Nrg-1 50 ng/ml
Control media
j
l
k
GFAP/DAPI
Fresh
Nrg-1 200 ng/ml
Incubated
Nrg-1 200 ng/ml
m
n
GFAP/DAPI
(i)
### Chart
| Category | |
|---|---|
| Microglia Media | 62.87577071379249 |
| Incubated Nrg-1 50 | 63.886870085724425 |
| FreshNrg-1 50 | 32.364549686556934 |
| Incubated Nrg-1 200 | 51.2449987659857 |
| Fresh Nrg-1 200 | 27.782812378204873 |****
*
****
Proportion of GFAP+/DAPI+ cells
normalized to control media
****
Fresh
Nrg-1 50 ng/ml
Incubated
Nrg-1 50 ng/ml
Control media
r
q
p
NG2/DAPI
Fresh
Nrg-1 200 ng/ml
Incubated
Nrg-1 200 ng/ml
s
t
NG2/DAPI
(o)
****
### Chart
| Category | |
|---|---|
| Control media | 23.8376021735206 |
| Incubated Nrg-1 50 | 27.87419636630416 |
| Fresh Nrg-1 50 | 43.54556818717864 |
| Incubated Nrg-1 200 | 32.3499343164675 |
| Fresh Nrg-1 200 | 46.31675842356486 |***
***
Proportion of NG2+/DAPI+ cells
normalized to control media
