# Supplementary material for: Neuregulin-1 Fosters Supportive Interactions between Microglia and Neural Stem/Progenitor Cells
Source: Stem Cells Int. 2019 Apr 7;2019:8397158. doi: 10.1155/2019/8397158 (PMC6476022; doi:10.1155/2019/8397158)
Supplement: Supplementary 5 — Supplementary Figure 5: evidence to verify that the original Nrg-1 treatment in MCM did not affect the outcomes of NPC mobilization. [file 8397158.f5.pptx]

## Slide 1
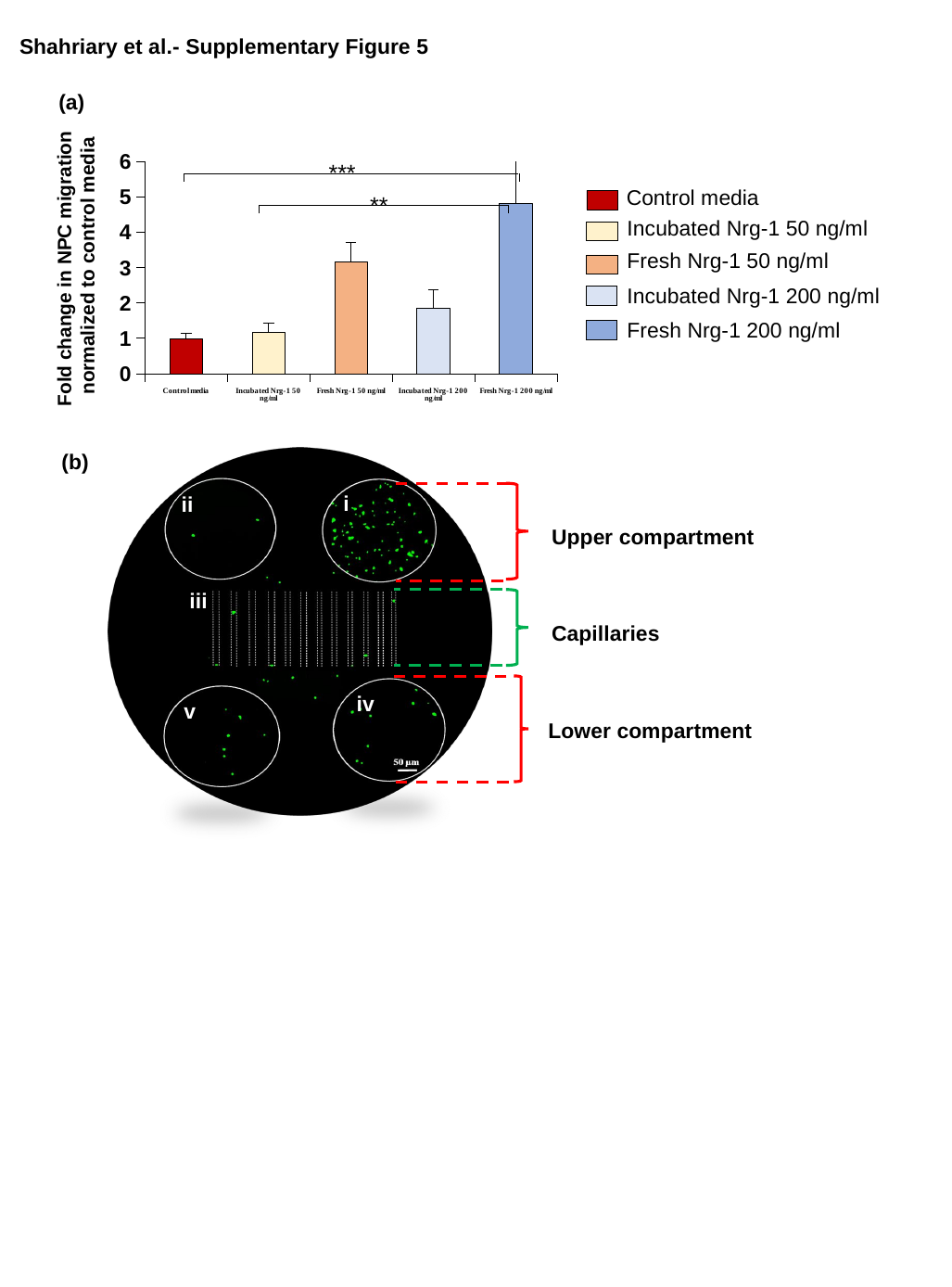

Shahriary et al.- Supplementary Figure 5
(a)
### Chart
| Category | |
|---|---|
| Control media | 1.0 |
| Incubated Nrg-1 50 ng/ml | 1.1851851851851851 |
| Fresh Nrg-1 50 ng/ml | 3.1666666666666665 |
| Incubated Nrg-1 200 ng/ml | 1.851851851851852 |
| Fresh Nrg-1 200 ng/ml | 4.822222222222222 |***
**
Fold change in NPC migration
 normalized to control media
Control media
Incubated Nrg-1 50 ng/ml
Fresh Nrg-1 50 ng/ml
Incubated Nrg-1 200 ng/ml
Fresh Nrg-1 200 ng/ml
(b)
i
ii
Upper compartment
iii
Capillaries
iv
v
Lower compartment
